# Synchronous Manipulation of Ion and Electron Transfer in Wadsley–Roth Phase Ti‐Nb Oxides for Fast‐Charging Lithium‐Ion Batteries

**DOI:** 10.1002/advs.202104530

**Published:** 2021-12-28

**Authors:** Yang Yang, Jingxin Huang, Zhenming Cao, Zeheng Lv, Dongzhen Wu, Zhipeng Wen, Weiwei Meng, Jing Zeng, Cheng Chao Li, Jinbao Zhao

**Affiliations:** ^1^ State Key Lab of Physical Chemistry of Solid Surfaces State‐Province Joint Engineering Laboratory of Power Source Technology for New Energy Vehicle College of Chemistry and Chemical Engineering Xiamen University Xiamen 361005 P. R. China; ^2^ School of Chemical Engineering and Light Industry Guangdong University of Technology Guangzhou 510006 P. R. China; ^3^ State Key Laboratory of Vanadium and Titanium Resources Comprehensive Utilization Panzhihua 617000 P. R. China

**Keywords:** electrostatic self‐assembly, fast‐charging lithium‐ion batteries, in situ XRD and Raman spectroscopy, solution combustion, TiNb_2_O_7_

## Abstract

Implementing fast‐charging lithium‐ion batteries (LIBs) is severely hindered by the issues of Li plating and poor rate capability for conventional graphite anode. Wadsley–Roth phase TiNb_2_O_7_ is regarded as a promising anode candidate to satisfy the requirements of fast‐charging LIBs. However, the unsatisfactory electrochemical kinetics resulting from sluggish ion and electron transfer still limit its wide applications. Herein, an effective strategy is proposed to synchronously improve the ion and electron transfer of TiNb_2_O_7_ by incorporation of oxygen vacancy and N‐doped graphene matrix (TNO_−_ *_x_* @N‐G), which is designed by combination of solution‐combustion and electrostatic self‐assembly approach. Theoretical calculations demonstrate that Li^+^ intercalation gives rise to the semi‐metallic characteristics of lithiated phases (Li*_y_* TNO_−_ *_x_*), leading to the self‐accelerated electron transport. Moreover, in situ X‐ray diffraction and Raman measurements reveal the highly reversible structural evolution of the TNO_−_ *_x_* @N‐G during cycling. Consequently, the TNO_−_ *_x_* @N‐G delivers a higher reversible capacity of 199.0 mAh g^−1^ and a higher capacity retention of 86.5% than those of pristine TNO (155.8 mAh g^−1^, 59.4%) at 10 C after 2000 cycles. Importantly, various electrochemical devices including lithium‐ion full battery and hybrid lithium‐ion capacitor by using the TNO_−_ *_x_* @N‐G anode exhibit excellent rate capability and cycling stability, verifying its potential in practical applications.

## Graphical abstract

An effective strategy is proposed to synchronously improve the ion and electron transfer of TiNb_2_O_7_ by incorporation of oxygen vacancy and N‐doped graphene matrix, which is designed by combination of solution‐combustion and electrostatic self‐assembly approach. Density functional theory calculations demonstrate that Li^+^ intercalation gives rise to the semi‐metallic characteristics of lithiated phases (Li*_y_* TNO_−_ *_x_*), leading to the self‐accelerated electron transport.

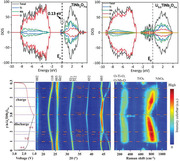

## 1 Introduction

Since the initial commercialization in the early 1990s, lithium‐ion batteries (LIBs) have been utilized as dominant power sources for ubiquitous portable electronic devices. With the incessant development, the state‐of‐the‐art LIBs have also allowed for the application in electric vehicles (EVs). However, range anxiety has been one of the most critical challenges for the competition between EVs and traditional internal combustion engines. The key to alleviate this anxiety is to develop fast charging battery technologies. Hence, the United States Department of Energy (DOE) proposed an interim goal to achieve a 15 min recharge time for high energy density batteries (≈180 Wh kg^−1^), the so‐called extreme fast charging (XFC).^[^ ^1^ ^]^ Nevertheless, the underlying issues of Li plating and particle cracking for conventional graphite anodes under high charging rates severely impede the enhancement of XFC performance.^[^ ^2^ ^]^ TiO_2_ has been widely investigated as one of high‐rate anode candidates due to its high theoretical capacity (≈335.4 mAh g^−1^ based on one electron transfer of Ti^4+^/Ti^3+^ redox couple) and safe lithiation potential (1.5–1.8 V vs Li^+^/Li). However, the sluggish electrochemical kinetics resulting from intrinsically low electronic conductivity (≈10^−12^ S cm^−1^) and Li^+^ diffusion coefficient (≈10^−15^ cm^2^ s^−1^) impede its practical application.^[^ ^3^ ^]^ Another promising high‐rate anode material is spinel Li_4_Ti_5_O_12_, which seems to be a more appropriate option for developing fast charging LIBs due to its moderate lithiation voltage (≈1.55 V vs Li^+^/Li) and characteristic “zero‐strain” structural evolution during (de)lithiation processes.^[^ ^4^ ^]^ Despite the above merits, the relatively low theoretical capacity (≈175 mAh g^−1^) limits its wide applications.

Recently, TiNb_2_O_7_ is emerging as compelling contenders to replacing Li_4_Ti_5_O_12_ due to its nearly two times higher theoretical capacity, which was first investigated by Goodenough's group at 2011.^[^ ^5^ ^]^ TiNb_2_O_7_ belongs to a typical Wadsley–Roth phase, which is consist of (3 × 3)_∞_ blocks extended infinitely in each plane.^[^ ^6^ ^]^ Crystallographically, the interconnected tunnel structure in TiNb_2_O_7_ along the *b* axis can potentially serve as multilane highways for facile and rapid Li^+^ ion diffusion.^[^ ^7^ ^]^ Furthermore, the Nb^5+^/Nb^3+^ redox couple with equilibrium potential of ≈1.6 V (vs Li^+^/Li) eliminates the risk of Li plating fundamentally.^[^ ^8^ ^]^ Therefore, TiNb_2_O_7_ has been extensively studied as one of the most promising anode candidates for fast‐charging LIBs.^[^ ^9^ ^]^ To get deeper insight into the electrochemical reaction, the intercalation of Li^+^ into TiNb_2_O_7_ includes three steps typically: 1) the transport of Li^+^ ions in the electrolyte; 2) the transfer of electron and simultaneous intercalation reaction at the interface; 3) Li^+^ ions diffusion in the bulk TiNb_2_O_7_, and every step may become the rate‐determining process to affect the electrochemical kinetics. The main obstacles hindering the use of TiNb_2_O_7_ in high‐rate applications still lie on the poor electronic conductivity and unsatisfactory apparent Li^+^ ion diffusion coefficients.

Targeting at the above‐mentioned drawbacks restraining the electrochemical kinetics, various modification strategies have been proposed. Constructing nanostructured or porous TiNb_2_O_7_ is a feasible approach to enhance Li^+^ ions transport through reducing the Li^+^ diffusion path and increasing the electrode/electrolyte contact area.^[^ ^10^ ^]^ However, previous synthesis methods containing hydrothermal reaction, electrospinning, and pulsed laser deposition are time‐consuming and complex, which are not suitable for large‐scale preparation.^[^ ^11^ ^]^ Besides, ensuring unobstructed electron transport in the whole electrode is also of great importance.^[^ ^12^ ^]^ Thus, carbon coating is frequently applied in nanostructured TiNb_2_O_7_ to further enhance the rate capability.^[^ ^13^ ^]^ Given that all, it is highly desirable to develop a simple and scalable route to simultaneously combine the advantages of nanoengineering and carbon coating technologies.

In this work, we developed a facile solution‐combustion method to synthesize nanoporous TNO, which was further encapsulated by nitrogen‐doped graphene layers via the fast electrostatic self‐assembly route. Unexpectedly, the reducing environment provided by the H_2_ and surrounding graphene around TNO nanocrystals also creates rich oxygen defects in the crystalline host. In the rationally designed TNO_−_ *_x_* @N‐G, N‐doped graphene serves as a highly conductive network for expeditious electron transfer, and rich oxygen defects in TNO nanocrystals provide sufficient active sites for Li^+^ insertion and diffusion. The synergistic enhancement of electron/ion transport endows the TNO_−_ *_x_* @N‐G rapid electrochemical kinetics, allowing it to be used under ultra‐high rate conditions (e.g., a high reversible capacity of 89.2 mAh g^−1^ at 100 C). Moreover, the TNO_−_ *_x_* @N‐G anode is also coupled with commercial LiFePO_4_ and active carbon cathodes to assemble electrochemical devices including lithium‐ion full battery and hybrid lithium‐ion capacitor, and their electrochemical performance is investigated comprehensively.

## 2 Results and Discussion

**Figure 1:**
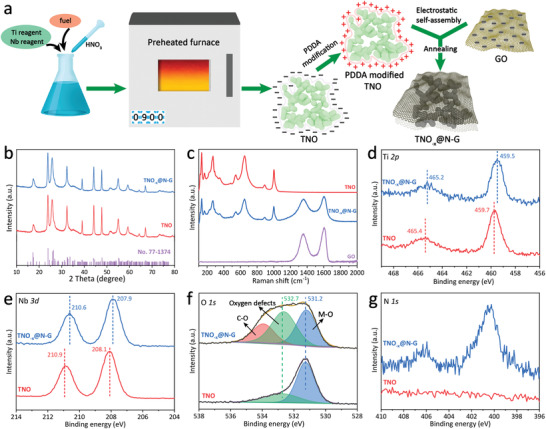
a) Schematic illustration of the synthesis of the TNO_−_ *_x_* @N‐G. b) XRD patterns of the TNO and TNO_−_ *_x_* @N‐G. c) Raman spectra of the TNO, TNO_−_ *_x_* @N‐G and GO. d–g) High‐resolution XPS spectra of the TNO and TNO_−_ *_x_* @N‐G: d) Ti 2p, e) Nb 3d, f) O 1s, and g) N 1s.

In this work, a simple one‐step solution combustion route has been employed to prepare porous nanocrystalline TNO (**Figure** **1**a). The reaction solution is composed of reactant and fuel, respectively. In the reactant part, [TiO(NO_3_)_2_] and Nb(HC_2_O_4_)_5_·*x*H_2_O act as the source of Ti and Nb in TiNb_2_O_7_, and the excessive HNO_3_ can not only prevent the hydrolysis of Nb^5+^ to form Nb(OH)_5_ precipitation but also trigger drastic combustion reactions when interacted with fuel owing to its strong oxidization. In this work, glycine was favored as the fuel by virtue of its unique features of excellent product uniformity owing to the complexing coordination effect between glycine and metal ions, avoiding selective precipitation when solvent quickly evaporates. As the system temperature rises to the ignition point, the self‐propagating combustion reaction occurs rapidly and can be completed within an extremely short duration of several minutes. The reaction is expected to occur according to the following empirical equation (Equation (1))

(1) $$\begin{array}{c} \begin{array}{c} \left[\mathrm{TiO}{\left(NO_{3}\right)}_{2}\right]\left(\mathrm{aq}\right)+2\mathrm{Nb}{\left(HC_{2}O_{4}\right)}_{5}\left(\mathrm{aq}\right)+xC_{2}H_{5}NO_{2}\left(\mathrm{aq}\right)+\left(2.5+2.25x\right)O_{2}\left(g\right)\\ \;\to \mathrm{TiN}b_{2}O_{7}\left(s\right)+\left(5+2.5x\right)H_{2}O\left(g\right)+\left(1+0.5x\right)N_{2}\left(g\right)+\left(20+2x\right)O_{2}\left(g\right) \end{array} \end{array}$$

As can be seen, the amount of gaseous products increases as more fuel (*x*) was added.^[^ ^14^ ^]^ Here, we choose *x* to be as high as 40. During the combustion process, the quick release of massive gases such as N_2_, CO_2_, and H_2_O leads to the formation of fine TNO particles with high porosity (Figure S1, Supporting Information).

The TNO_−_ *_x_* @N‐G composite was further synthesized via an electrostatic self‐assembly route. After modified with PDDA (poly dimethyl diallyl ammonium chloride), the surface charge of TNO nanoparticles is successfully converted from negatively charged (zeta potential = −19.71 mV) to positively charged (zeta potential = 43.99 mV). When the positively charged TNO‐PDDA aqueous dispersion is mixed with negatively charged GO dispersion (zeta potential = −35.56 mV), TNO nanoparticles will be encapsulated in flexible GO layers due to the spontaneous electrostatic interaction. After annealing under the H_2_/Ar atmosphere, the residual PDDA acting as N source leads to the formation of N‐doped graphene. Meanwhile, the reduced atmosphere also creates advantageous oxygen defects in the crystalline host of TiNb_2_O_7_. Finally, TNO nanoparticles with oxygen defects encapsulated in N‐doped graphene layers (TNO_−_ *_x_* @N‐G) can be obtained.

X‐ray diffraction (XRD) measurements were performed to study the crystallinity of the as‐prepared TNO and TNO_−_ *_x_* @N‐G (Figure 1b). All XRD peaks of both the TNO and TNO_−_ *_x_* @N‐G samples match well with the standard pattern of monoclinic crystalline TiNb_2_O_7_ (space group: *C2/m*, JCPDS No. 77–1374) without obvious impurity phases (such as Nb_2_O_5_ and TiO_2_) being detected.^[^ ^15^ ^]^ Raman spectra of the TNO, TNO_−_ *_x_* @N‐G and GO are also shown in Figure 1c. The peaks of TNO and TNO_−_ *_x_* @N‐G less than 1200 cm^−1^ match well with the characteristic peaks of previously reported TiNb_2_O_7_.^[^ ^16^ ^]^ The two peaks located at 998.1 and 892.2 cm^−1^ correspond to the vibrations of the edge‐ and corner‐shared NbO_6_ octahedron, respectively. Another two peaks located at 649.7 and 539.1 cm^−1^ can be attributed to the vibrations of the edge‐ and corner‐shared TiO_6_ octahedron, respectively. The peaks located at 270.0 and 169.1 cm^−1^ correspond to vibrations of complex models involving the antisymmetric and symmetric bending vibrations of O—Nb—O and O—Ti—O. And the peak observed at 134.7 cm^−1^ is originated from the external motions based on the M—O stretching.^[^ ^17^ ^]^ Another two peaks of TNO_−_ *_x_* @N‐G at around 1358 and 1601 cm^−1^ are the characteristic D band and G band of carbonaceous materials (rGO), which belongs to the vibrations of disorder carbon and ordered graphitic carbon, respectively.^[^ ^18^ ^]^ The peak intensity ratio of D band to G band (*I*_D_/*I*_G_) of the TNO_−_ *_x_* @N‐G (0.91) is higher than that of GO (0.81), indicating that GO is successfully reduced after annealing.^[^ ^19^ ^]^ Furthermore, the carbon content of the TNO_−_ *_x_* @N‐G composite was estimated to be ≈7.7 wt% from the thermogravimetric analysis (Figure S2, Supporting Information).

The chemical environment and valence state of the TNO and TNO_−_ *_x_* @N‐G samples were further examined by X‐ray photoelectron spectroscopy (XPS, Figure 1d–g), and all spectra were calibrated by the C 1s peak at 284.8 eV. For the Ti 2p XPS spectrum of pristine TNO (Figure 1d), two strong peaks located at 465.4 and 459.7 eV contribute to Ti 2p 1/2 and Ti 2p 3/2, respectively. While these two peaks of TNO_−_ *_x_* @N‐G shift to lower binding energies of 465.2 and 459.5 eV, suggesting that a small amount of Ti^4+^ has been reduced to the lower valence of Ti^3+^ during the annealing process. For the Nb 3d XPS spectrum of pristine TNO (Figure 1e), two peaks at 210.9 and 208.1 eV correspond to Nb 3d 3/2 and Nb 3d 5/2, respectively. It is obvious that the binding energies of TNO_−_ *_x_* @N‐G also shift to lower values compared with that of TNO, corroborating the reduction effect. Although both Nb^5+^ and Ti^4+^ yield semi‐conductive materials with poor electronic conductivity, the reduced valence of Ti^3+^ and Nb^4+^ will increase electron density in 4d orbits, leading to the enhanced electron transport in the bulk TiNb_2_O_7_. The reduction of metal atoms in oxides is usually accompanied by the introduction of oxygen defects, which is confirmed by the O 1s XPS spectra (Figure 1f). The obvious peak located at 531.2 eV is assigned to the Ti/Nb—O bond, and another broad peak located ≈532.7 eV should be contributed to the oxygen deficient region. It can be clearly seen that the peak intensity of oxygen vacancy in the TNO_−_ *_x_* @N‐G is much higher than that in pristine TNO, indicating the existence of rich oxygen deficiency. To further verify the defect chemistry, electron paramagnetic resonance spectra were also obtained (Figure S3, Supporting Information). The TNO_−_ *_x_* @N‐G shows a much stronger symmetrical signals at *g* = 1.991 (the signal of unpaired electrons at oxygen vacancy sites) than that of TNO, confirming the existence of rich oxygen defects. Additionally, obvious N 1s signal is also observed in the TNO_−_ *_x_* @N‐G (Figure 1g), which is derived from the self‐doping of graphene with PDDA as the nitrogen source. The nitrogen dopants in graphene layer endow carbon atoms more electronegativity, which can promote the adsorption and diffusion of Li^+^ ions.

**Figure 2:**
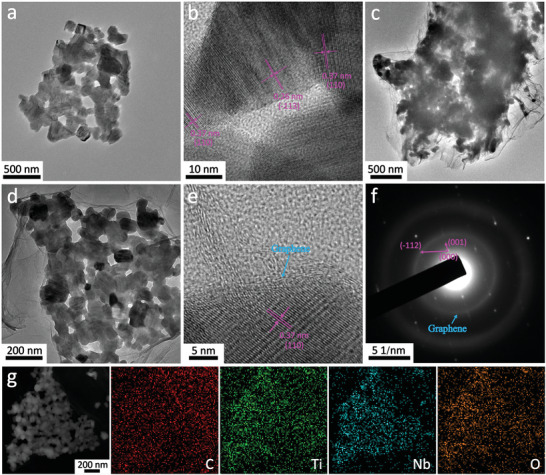
a) TEM and b) HR‐TEM images of the TNO. c,d) TEM and e) HR‐TEM images of the TNO_−_ *_x_* @N‐G. f) SADE patterns of the TNO_−_ *_x_* @N‐G. g) High‐angle annular dark‐field image and corresponding elemental mapping of the TNO_−_ *_x_* @N‐G.

The micro‐morphologies of the TNO and TNO_−_ *_x_* @N‐G were investigated by TEM (transmission electron microscopy), as shown in **Figure** **2**. The low‐magnification TEM image of TNO exhibits an agglomeration of ultrafine nanoparticles with an average size distribution of ~60.3 nm (Figure 2a and Figure S4, Supporting Information). The nanoporous structure can be observed among TNO nanoparticles, which should be induced by the drastic release of gasses during the combustion process. As shown in Figure 2b, these nanoparticles are highly crystalline, which shows distinct lattice fringes with interplanar spaces of 0.37 and 0.26 nm, corresponding to the (110) and (213) planes of monoclinic TiNb_2_O_7_, respectively. For the TNO_−_ *_x_* @N‐G, TNO nanocrystals are well encapsulated by thin crumpled graphene sheets (Figure 2c,d). It can be clearly observed that graphene sheets are tightly anchored to the surface of TNO in the high‐resolution TEM (HR‐TEM) image (Figure 2e). The TNO nanoparticles inside the composite material show clear lattice space of around 0.37 nm, which is in good agreement with the (110) plane of TNO. The layers outside the TNO nanoparticles with irregular lattice fringes should be graphene layers. The SAED (selected area electron diffraction) patterns of TNO_−_ *_x_* @N‐G further confirm that the TNO nanocrystals are single crystalline, and the faintness diffraction rings are assigned to the stacks of graphene (Figure 2f). The energy‐dispersive X‐ray spectroscopy mapping result (Figure 2g) indicates that Ti, Nb, and O elements are uniformly spread in the C, demonstrating a homogenous distribution of the TNO nanoparticles in the conductive graphene framework.

**Figure 3:**
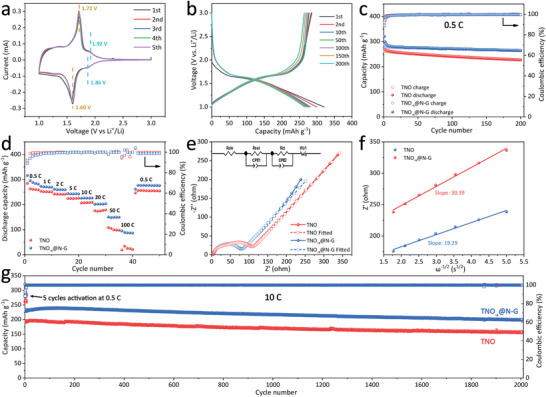
a) CV curves of the TNO_−_ *_x_* @N‐G electrode for the first five cycles. b) Typical charge and discharge profiles of the TNO_−_ *_x_* @N‐G at 0.5 C. c) Cycling performance of the TNO and TNO_−_ *_x_* @N‐G at 0.5 C. d) Rate performance of the TNO and TNO_−_ *_x_* @N‐G. e) EIS of the TNO and TNO_−_ *_x_* @N‐G electrodes. f) Dependence of the real impedance (*Z*) on the reciprocal of the square root of the frequency (*υ* ^−1/2^) for determination of the Warburg coefficient (*σ*). g) Long‐term cycling performance of the TNO and TNO_−_ *_x_* @N‐G at 10 C.

CR‐2016‐type half‐cells were assembled to evaluate the Li^+^ storage performance of the TNO and TNO_−_ *_x_* @N‐G (**Figure** **3**). The initial three cyclic voltammetry (CV) curves of the TNO_−_ *_x_* @N‐G between 1.0 and 3.0 V (vs Li^+^/Li) at the scan rate of 0.1 mV s^−1^ are shown in Figure 3a, which are similar to the previous reports.^[^ ^10^, ^11^, ^15^ ^]^ The broad shoulder peaks at ≈1.86 V (cathodic) and 1.92 V (anodic) can be assigned to the Ti^4+^/Ti^3+^ redox couple. The obvious pair of reduction/oxidation peaks located at 1.60/1.72 V can be attributed to the pair of Nb^5+^/Nb^4+^. The broad bump between 1.0 and 1.4 V may correspond to the Nb^4+^/Nb^3+^. Furthermore, the CV curves after the initial cycle coincide well, indicating the good reversibility of electrochemical redox reactions. Figure 3b shows the charging and discharging profiles of the TNO_−_ *_x_* @N‐G at 0.5 C. The TNO_−_ *_x_* @N‐G delivers initial discharge/charge capacities of 285.3 and 320.5 mAh g^−1^ with an initial Columbic efficiency (CE) of 89.0%. The capacity contribution of graphene in the TNO_−_ *_x_* @N‐G is nearly negligible (Figure S5, Supporting Information). The corresponding capacities for the TNO are 284.5 and 262.1 mAh g^−1^ with the initial CE of 92.1% (Figure S6, Supporting Information). The enhanced reversible capacity of TNO_−_ *_x_* @N‐G should be associated with the more electrochemically active sites created by oxygen vacancies, and the slightly reduced CE may be induced by the irreversible capacity loss of graphene.^[^ ^11^, ^20^ ^]^ It should be noticed that no obvious change is observed for the voltage curves of the TNO‐rGO composite during cycling, demonstrating the high cyclic stability and small polarization of the TNO_−_ *_x_* @N‐G electrode. Figure 3c shows the cycling performance of the TNO and TNO_−_ *_x_* @N‐G at 0.5 C. A high reversible capacity of 264.8 mAh g^−1^ can be retained after 200 cycles for the TNO_−_ *_x_* @N‐G, corresponding to the capacity retention of 92.8%. Obviously, the capacity retention is better than that of the TNO (86.6%). XPS measurements of the TNO and TNO_−_ *_x_* @N‐G electrodes (Figure S7, Supporting Information) after ten cycles show that the binding energies of Ti 2p and Nb 3d for the TNO_−_ *_x_* @N‐G are still slightly lower than those for the TNO, implying the existence of oxygen vacancies after cycling.

Rate performance of the TNO and TNO_−_ *_x_* @N‐G was also investigated (Figure 3d). The TNO_−_ *_x_* @N‐G exhibits the average capacity of 287.6 mAh g^−1^ at 0.5 C. At higher current densities of 1 C, 2 C, 5 C, 10 C, 20 C, and 50 C, the average charge capacities are 270.8, 259.9, 243.0, 226.0, 202.3, and 149.5 mAh g^−1^, respectively. Even at an extremely high current density of 100 C, a considerably high capacity of 89.2 mAh g^−1^ can be still maintained. For the pristine TNO, it shows average capacities of 265.7, 251.8, 241.7, 224.8, 207.8, 176.0, 102.0, and 25.1 mAh g^−1^ as the current densities rising from 0.5 C to 100 C. It is clear that the average capacity retention of the TNO_−_ *_x_* @N‐G during high rate cycling is better than that of TNO. It should be noticed that the TNO_−_ *_x_* @N‐G also shows much better rate capability compared with commercial Li_4_Ti_5_O_12_ (Figure S8, Supporting Information). To further study the cyclic performance of the TNO_−_ *_x_* @N‐G, a long‐term cycling test at 10 C was performed (the cells were previously activated at a lower current density of 0.5 C for five cycles). As shown in Figure 3g, the TNO only maintains a much lower capacity of 155.8 mAh g^−1^ with the capacity retention of 59.4% after 2000 cycles. While the capacity of TNO_−_ *_x_* @N‐G still stabilizes at a remarkably high capacity of 199.0 mAh g^−1^ with a capacity retention of 86.5%. The outstanding cycling stability is comparable to other previously reported TiNb_2_O_7_‐based materials (Table S1, Supporting Information). To further verify the importance of N‐doped graphene, the TNO_−_ *_x_* sample with oxygen defects but without graphene was prepared by annealing pristine TNO at 700 °C under the H_2_/Ar atmosphere. The reversible capacity of the TNO_−_ *_x_* electrode at 0.5 C is close to the value of TiNb_2_O_7_@N‐G, but which is lower than that of TiNb_2_O_7_@N‐G at 10 C. The evident capacity gap at the higher current density demonstrates that the improved electrochemical performance by creating oxygen defects singly is inferior to the combined strategy of graphene hybridization and oxygen vacancies (Figure S9, Supporting Information). Because the well‐established conduction framework of N‐doped graphene in the whole electrode is favorable for the improvement of the cyclic stability (Figure S10, Supporting Information).

The electrochemical impedance spectra (EIS) tests for both electrodes are carried out at the fully delithiated state after five cycles to probe the mechanism of the enhanced electrochemical performance of the TNO_−_ *_x_* @N‐G (Figure 3e). Every EIS plot is composed of a compressed semi‐circle and an inclined line, which is related to the interfacial charge‐transfer resistance and the Li^+^ ions diffusion process in the electrodes, respectively.^[^ ^21^ ^]^ The compressed semi‐circle includes two overlapped semi‐circles which represent the surface film resistance (*R*_sei_) and charge‐transfer resistance for Li^+^ ions intercalation (*R*_ct_), respectively.^[^ ^22^ ^]^ The EIS plots are fitted via the equivalent circuit (inset in Figure 3e) and the related parameters are shown in Table S2 in the Supporting Information. It can be clearly seen that the interfacial charge‐transfer resistance of the TNO_−_ *_x_* @N‐G electrode (70 Ω) is obviously smaller than that of the TNO electrode (95.04 Ω). Besides, the powder electronic conductivity measurements were also carried out at the pressure of 4.0 MPa (Table S2, Supporting Information). The electronic conductivity of the TNO_−_ *_x_* @N‐G (1.38 S cm^−1^) is enhanced by about five orders of magnitude compared with the TNO (3.73 × 10^−5^ S cm^−1^). The results demonstrate that the highly conductive N‐doped graphene matrix can effectively enhance the electron transport and decrease the interfacial charge‐transfer resistance.^[^ ^23^ ^]^ Moreover, The EIS plots at low‐frequency region can be used to compare the Li^+^ ion diffusion coefficient (*D*_Li_) using the following equation

(2) $$Z_{\mathrm{real}}=R_{\mathrm{ct}}+R_{\mathrm{sei}}+\sigma \omega^{-1/2}$$

(3) $$D_{\mathrm{Li}}=\frac{R^{2}T^{2}}{2A^{2}n^{4}F^{4}{C_{\mathrm{Li}}}^{2}\sigma^{2}}$$

where *R* is the gas constant, *T* is the absolute temperature, *A* is the surface area, *n* is the transferred electrons number, *F* is the Faraday's constant, and *C*_Li_ is the concentration of Li^+^ ions in solid. It can be deduced that these values are fixed in the TNO and TNO_−_ *_x_* @N‐G electrodes, and *D*_Li_ is directly related to *σ*. *σ* is the Warburg factor, which can be obtained by using the linear relationship between *Z*_real_ and *ω* ^−1/2^. As shown in Figure 3f, *σ* of the TNO_−_ *_x_* @N‐G electrode is calculated to be 19.29, which is smaller than that of the TNO electrode (30.39). It is obvious that *D*_Li_ of the TNO_−_ *_x_* @N‐G electrode is 2.5 times higher than that of the TNO electrode, which may be due to the existence of oxygen defects. Moreover, $D_{Li^{+}}$ of the TNO_−_ *_x_* @N‐G electrode is also measured by galvanostatic intermittent titration technique (Figure S11, Supporting Information). As shown in Table S3 in the Supporting Information, the calculated apparent $D_{Li^{+}}$ of the TNO_−_ *_x_* @N‐G electrode is in the order of 10^−9.5^ to 10^−10.5^ cm^2^ s^−1^, which is higher than those of previously reported Li_4_Ti_5_O_12_ and TiO_2_. The enhanced charge‐transfer process and higher Li^+^ ion diffusion coefficient of the TNO_−_ *_x_* @N‐G electrode should lead to improved electrochemical kinetics, contributing to the outstanding rate capability and good cycling stability.

**Figure 4:**
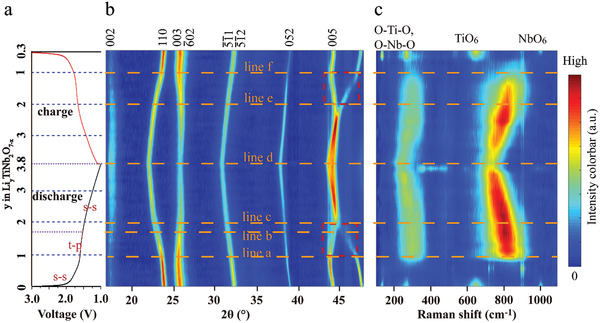
In situ electrochemical XRD patterns of the TNO_−_ *_x_* @N‐G electrode during the initial discharge/charge cycle: a) the corresponding voltage profiles of the TNO_−_ *_x_* @N‐G obtained at 19 mA g^−1^ (the *y* in “Li*_y_* TiNb_2_O_7_” is calculated based on the TiNb_2_O_7−_ *_x_* quantity in the TNO_−_ *_x_* @N‐G). b) The simultaneous in situ electrochemical XRD patterns corresponding to (a) with the arbitrary unit (a.u.) colorbar on the right indicating the increasing intensity from blue to red. The red dashed rectangles in (b) indicate the regions which exhibit the distinct difference of the structural evolution in the discharging and charging. c) The simultaneous in situ electrochemical Raman spectra corresponding to (a) with the arbitrary unit (a.u.) colorbar on the right indicating the increasing intensity from blue to red. The dashed lines in (b) and (c) indicate the different reaction stages of TNO_−_ *_x_* @N‐G in the initial discharging/charging processes. A part of the single XRD patterns and Raman spectra extracted from the (b) and (c) is shown in Figure S12 in the Supporting Information.

The structural evolution of the TNO_−_ *_x_* @N‐G in the initial discharging/charging cycle was investigated by in situ XRD and Raman spectroscopy to establish the structure–performance relationship, which can help to understand the intrinsic electrochemical reaction to further improve the electrochemical performance of this material rationally (**Figure** **4**). In Figure 4a, from the pristine state to the line a (*y* = 0–1.0) in the discharging, the galvanostatic curve shows a sloped voltage curve. Correspondingly, the XRD peaks continuously shift to lower 2*θ* value in Figure 4b, indicating the lattice expansion of the TiNb_2_O_7−_ *_x_*_._ Thus, the electrochemical and XRD tests indicate a solid solution process. In the discharging between the line a and b (*y* = 1.0–1.7), the discharging curve exhibits a small plateau while the XRD peaks show a significant intensity decrease, which was thought to be a region of two phase co‐existence in the previous reports.^[^ ^20^ ^]^ From the Li*_y_* TiNb_2_O_7−_ *_x_* (*y* = 1.7) to the end of the discharging between the line b and the line d, the electrochemical and XRD tests also exhibit a solid solution process by the sloped voltage curve and the continuous 2*θ* angle shift of the XRD peaks.

**Figure 5:**
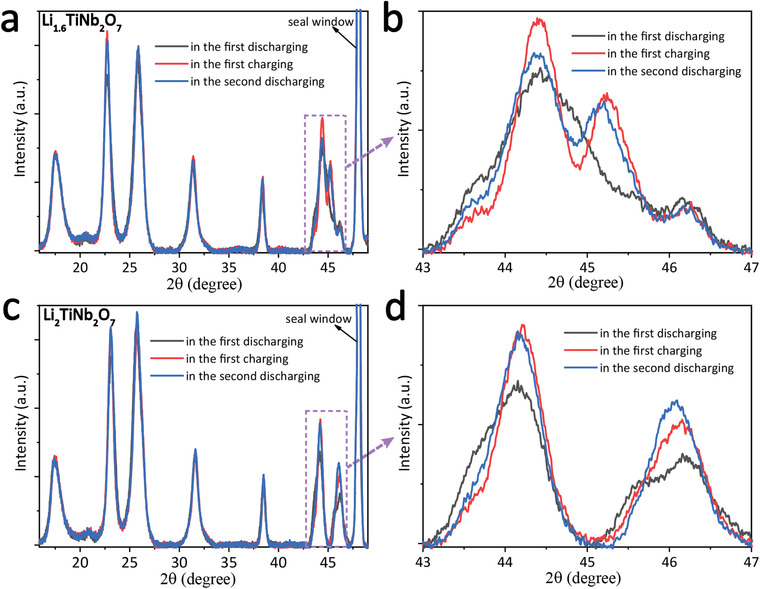
Ex situ XRD patterns of Li*_y_* TiNb_2_O_7_ for a) *y* = 1.6 and c) *y* = 2.0 in the first discharging, the first charging, and the second charging processes. The green dashed rectangles indicate the same 2*θ* angle range with that indicated by the red dashed rectangles in Figure 4b. (b) and (d) correspond to the purple dashed rectangles in (a) and (c).

In the charging of Li*_y_* TiNb_2_O_7−_ *_x_* (*y* = 3.8–2.0), from the line d to the line e, the XRD pattern exhibits a reverse evolution process with that in the discharging process. However, in the region of Li*_y_* TiNb_2_O_7−_ *_x_* (*y* = 2.0–1.0) between the line e and the line f as the red dashed rectangles indicated in Figure 4b, the XRD pattern exhibits a distinct difference in the structural evolution from that in the discharging. From the XRD patterns extracted from the region in the red dashed rectangles in Figure 4b and Figure S13 in the Supporting Information, the XRD peak profile in the discharging exhibits a lower intensity and broader width than that in the charging, which may indicate an inhomogenous distribution of the strain in the lattice expansion process. In **Figure** **5**, the ex situ XRD test for the stages of the Li_1.6_TiNb_2_O_7−_ *_x_* (Figure 5a,b) and Li_2.0_TiNb_2_O_7−_ *_x_* (Figure 5c,d) in the first charging and the second discharging show nearly the same XRD peak profile, which may mean the structure of the material reached a stable state after the initial discharging process. The above results indicate that the TNO_−_ *_x_* @N‐G may undergo a structural activation in the first discharging process and become stable during the subsequent cycles.

From Figure 4c, the asymmetric profiles of the in situ electrochemical Raman spectra between the discharging and the charging were observed, which may be also related to the structural activation in the discharging due to the superior sensitivity of Raman spectroscopy to the change of the local M–O coordination structure of the material. Meanwhile, the asymmetric profile of the in situ Raman spectral change may also be related to the asymmetric Li^+^ storage performance between the discharging and the charging proposed by Tang et al.^[^ ^10e^ ^]^ In Figure S14a in the Supporting Information, some XRD peaks after the discharging/charging cycle exhibit lower intensity than that of the pristine state, which indicates an irreversible structural change after the discharging/charging cycle. The irreversibility of the structure of the material after the first cycle may be an important cause for the <100% initial CE in the initial cycle of the material. In Figure S14b in the Supporting Information, the Raman spectra after the discharging/charging cycle is also different from that of the pristine state, which further indicates the irreversible structural change after the discharging/charging cycle. Especially, the Raman spectral peaks related with the NbO_6_ exhibit a lower relative intensity than those related with the TiO_6_ after the first cycle, which may indicate the irreversibility of the NbO_6_. The local structural irreversibility agrees with the result indicated by the SAED in the previous work.^[^ ^24^ ^]^ Ex situ XRD and in situ Raman results (Figures S15 and S16, Supporting Information) of the TNO exhibit a similar structural evolution profile with that of TNO_−_ *_x_* @N‐G, demonstrating that oxygen vacancies and graphene can mainly improve the electrochemical activity of the TNO rather than affecting the solid phase Li^+^ storage mechanism of TNO.

**Figure 6:**
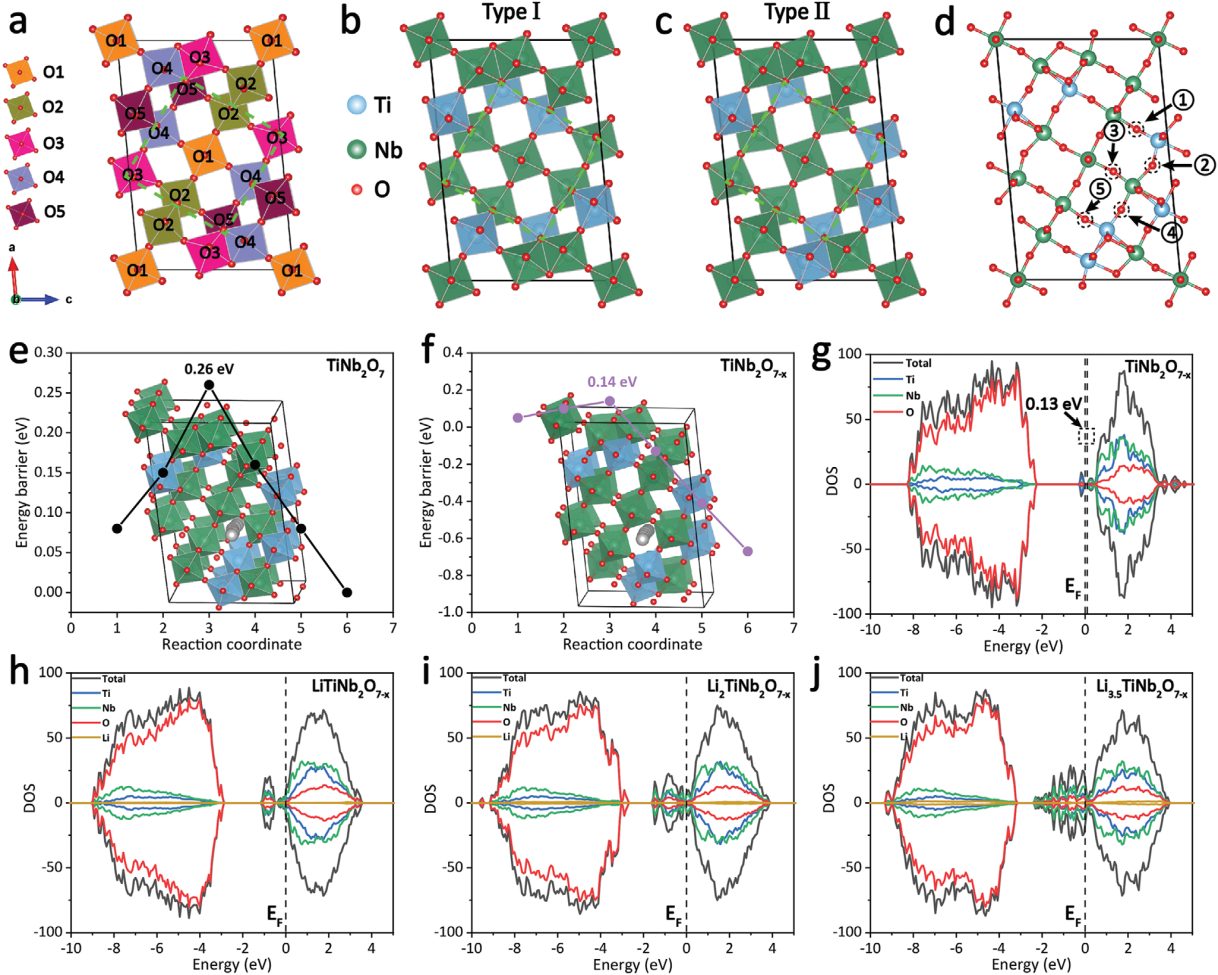
a) Wadsley–Roth crystallographic shear structure of TiNb_2_O_7_ with five chemically different octahedral sites (O1–O5). b,c) Two typical crystal structure models of TiNb_2_O_7_. d) Types of oxygen vacancy sites present in TiNb_2_O_7−_ *_x_*. Li^+^ diffusion energy barriers of e) TiNb_2_O_7_ and f) TiNb_2_O_7−_ *_x_* (inset is the diffusion path). g–j) DOS of TiNb_2_O_7−_ *_x_* and Li*_y_* TiNb_2_O_7−_ *_x_* phases.

Density functional theory (DFT) calculations of TiNb_2_O_7_ and TiNb_2_O_7−_ *_x_* were conducted to get deeper insight into their diversity in Li^+^ diffusion and electronic conduction (**Figure** **6**). Building accurate crystalline structure model of TiNb_2_O_7_ is a prerequisite for performing reliable theoretical calculations. TiNb_2_O_7_ crystallizes in space group *C2/m*, and is composed of 3 × 3 × ∞ ReO_3_‐type blocks connected by the edge‐sharing in each plane infinitely, where 3 × 3 represents that there are three MO_6_ octahedra length and three MO_6_ octahedra width, respectively (green dashed rectangle in Figure 6a). Some previous studies proposed that TiO_6_ and NbO_6_ octahedra are randomly distributed in the block due to the close ionic radii of Ti^4+^ (0.61 Å) and Nb^5+^ (0.64 Å).^[^ ^25^ ^]^ Actually, these octahedral sites in the structure of TiNb_2_O_7_ are crystallographically different, which can be distinguished into five types (O1–O5, Figure 6a).^[^ ^16b^ ^]^ Therefore, the completely disordered distribution of TiO_6_ and NbO_6_ octahedra is unreasonable based on the principle of thermodynamic stability. Cheetham and Von Dreele found that metal atoms with higher valance prefer the center of the blocks, while metal atoms with lower valance occupy the corner sites within niobium oxide blocks structures, and the nature of this ordering could be understood in terms of Coulombic interactions.^[^ ^26^ ^]^ To simplify the structural model of TiNb_2_O_7_, we assumed that the Ti occupancy in the corner of the block (O5) is 100%, and the Nb occupancy in the center of the block (O1) is 100%. Accordingly, there is only one remaining Ti atom undetermined in the block, which may be located at O2, O3, or O4 sites. Our DFT calculation results show that the relative energies of type I (one Ti atom in O2 site, Figure 6b), type II (one Ti atom in O3 site, Figure 6c), and type III (one Ti atom in O4 site, Figure S17, Supporting Information) are 0, −0.664, and −0.342 eV, respectively. Therefore, the type II crystalline structure of TiNb_2_O_7_ is preferred by following the Hohenberg–Kohn principle of the minimum ground‐state energy. Furthermore, the oxygen vacancy site in the block of the TiNb_2_O_7−_ *_x_* sample was also confirmed by the similar principle (Figure 6d). It can be seen that the relative energy of Type ⑤ oxygen vacancy site (−0.491 eV) is much lower than that of Type ① (0 eV), Type ② (0.124 eV), Type ③ (0.088 eV), and Type ④ (0.222 eV).

Based on the rationally established crystalline models of TiNb_2_O_7_ and TiNb_2_O_7−_ *_x_*, the Li^+^ diffusion behavior perpendicular to the block plane is investigated (insets in Figure 6e,f), which is generally accepted to be the most facile diffusion paths in Wadsley–Roth phase materials.^[^ ^27^ ^]^ Since the calculated energy barrier of Li^+^ down the tunnel in TiNb_2_O_7−_ *_x_* (0.14 eV, Figure 6e) is lower than that of TiNb_2_O_7_ (0.26 eV, Figure 6f), Li^+^ diffusion is energetically more favorable in TiNb_2_O_7−_ *_x_* than TiNb_2_O_7_. It can be explained that the electrostatic interaction between intercalated Li^+^ and terminal oxygen atoms of MO_6_ octahedra mainly contributes to the diffusion energy barrier, which should be reduced effectively through the introduction of oxygen vacancies. The density of states (DOS) of the corresponding crystal models were also calculated by DFT to evaluate the electronic structures (Figure 6g and Figure S18, Supporting Information). The result indicates that pure TiNb_2_O_7_ is a semiconductor and that its band gap is about 2.15 eV (Figure S18, Supporting Information). However, the band gap of TiNb_2_O_7−_ *_x_* is significantly reduced to only 0.13 eV (Figure 6g), validating that the electronic conductivity of TiNb_2_O_7−_ *_x_* is much better than the TiNb_2_O_7_. Interestingly, the Fermi level of the lithiated phase of LiTiNb_2_O_7−_ *_x_* shifts to the conduction band (Figure 6h), demonstrating the metallic character after Li^+^ insertion. After the intercalation of Li^+^ into TiNb_2_O_7−_ *_x_*, the overlap of d orbital between the edge‐sharing MO_6_ octahedra increases. And the edge‐sharing bonds filled with t2g orbitals of electrons provide an electronic conductivity transport route through the length of the LiTiNb_2_O_7−_ *_x_* crystal.^[^ ^28^ ^]^ Moreover, the higher lithiation degree of Li*_y_* TiNb_2_O_7−_ *_x_* further leads to the increased electron density close to the Fermi level (Figure 6i,j), revealing that the electronic conductivity of Li*_y_* TiNb_2_O_7−_ *_x_* is continuously enhanced during the lithiation process.

**Figure 7:**
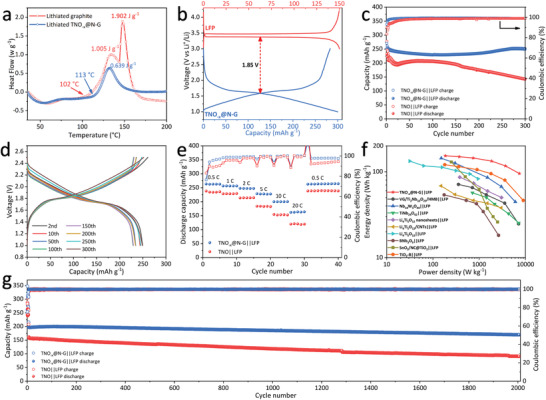
a) Differential scanning calorimetry traces of the lithiated graphite and the TNO_−_ *_x_* @N‐G containing electrolyte. b) Charge–discharge profiles of the TNO_−_ *_x_* @N‐G anode and LFP (LiFePO_4_) cathode at 0.5 C. c) Cycling performance and d) typical charge–discharge profiles of the TNO||LFP and TNO_−_ *_x_* @N‐G||LFP cells at 1 C. e) Rate performance of the TNO||LFP and TNO_−_ *_x_* @N‐G||LFP cells. f) Ragone plots of the TNO_−_ *_x_* @N‐G||LFP cell compared with other full batteries by using LFP as the cathode and Ti/Nb‐based materials as the anode. g) Long‐term cycling performance of the TNO||LFP and TNO_−_ *_x_* @N‐G||LFP cells at 10 C.

To further verify the practicability, lithium‐ion full batteries with the TNO and TNO_−_ *_x_* @N‐G electrodes as anodes were fabricated and evaluated (**Figure** **7**). The thermal stability of the lithiated anodes in the electrolyte is one of the most significant factors to make quantitative predictions about the safety of practical LIBs, and we selected the commercial graphite anode as a contrast (Figure 7a).^[^ ^29^ ^]^ In the case of the lithiated graphite (cut‐off voltage: 0.01 V vs Li^+^/Li), the thermal degradation began at around 102 °C which results in two exothermal reactions because of solid electrolyte interface (SEI) breakdown and lithium removal from the graphitic layers.^[^ ^30^ ^]^ However, for the lithiated TNO_−_ *_x_* @N‐G (cut‐off voltage: 1.0 V vs Li^+^/Li), there is only one exothermic reaction occurred at about 113 °C due to a much less amount of SEI formed. Moreover, the total heat of the lithiated TNO_−_ *_x_* @N‐G (0.639 J g^−1^) is much reduced compared with that of the lithiated graphite (2.907 J g^−1^), which should be beneficial to improve the thermal stability of the battery at high temperature.

By matching with the LFP (LiFePO_4_) cathode, the TNO_−_ *_x_* @N‐G||LFP full cell can output a considerable working voltage of ≈1.85 V (Figure 7b). The as‐fabricated TNO_−_ *_x_* @N‐G||LFP cell achieves an initial discharge capacity of 252.4 mAh g^−1^, and a high reversible capacity of 249.3 mAh g^−1^ can be retained after 300 cycles at 1 C, corresponding to the remarkable capacity retention of 98.8% (Figure 7c). In sharp contrast, the TNO||LFP cell only exhibits a much lower discharge capacity of 141.9 mAh g^−1^ with an unsatisfactory capacity retention of 61.0% after 300 cycles at 1 C. Impressively, the charge–discharge profiles of the TNO_−_ *_x_* @N‐G||LFP during different cycles mainly overlap without detectable voltage drop (Figure 7d), revealing the highly reversible electrochemical reactions in this battery prototype.

Rate performance of the TNO||LFP and TNO_−_ *_x_* @N‐G||LFP cells was also tested (Figure 7e). The TNO_−_ *_x_* @N‐G||LFP full cell exhibits the average capacity of 262.8 mAh g^−1^ at 0.5 C. At higher current densities of 1 C, 2 C, 5 C, 10 C, and 20 C, the average capacities are 256.3, 247.5, 227.7, 200.0, and 162.7 mAh g^−1^, respectively. Moreover, when the current density decreases to 0.5 C, the discharge capacity immediately recovers to as high as 262.7 mAh g^−1^, demonstrating the excellent tolerance against high‐rate cycling. For the contrastive TNO||LFP cell, it shows lower average capacities of 235.1, 228.6, 213.7, 183.3, 153.2, and 119.8 mAh g^−1^ as the current densities rise from 0.5 C to 100 C. Ragone plots of the TNO_−_ *_x_* @N‐G||LFP cell compared with other full batteries by using LFP as the cathode and Ti/Nb‐based materials as the anode are shown in Figure 7f. The TNO_−_ *_x_* @N‐G||LFP cell delivers the highest energy density of 153.5 Wh kg^−1^, which is obviously higher than the VG/Ti_2_Nb_10_O_29_/HMB||LFP (71.2 Wh kg^−1^),^[^ ^31^ ^]^ Nb_14_W_3_O_44_||LFP (143.8 Wh kg^−1^),^[^ ^32^ ^]^ TiNb_24_O_62_||LFP (56.9 Wh kg^−1^),^[^ ^33^ ^]^ Li_4_Ti_5_O_12_ nanosheets||LFP (86.4 Wh kg^−1^),^[^ ^34^ ^]^ Li_4_Ti_5_O_12_/CNTs||LFP (68.5 Wh kg^−1^),^[^ ^35^ ^]^ Li_4_Ti_5_O_12_||LFP (133 Wh kg^−1^),^[^ ^36^ ^]^ BNb_3_O_9_||LFP (53.8 Wh kg^−1^),^[^ ^37^ ^]^ SnO_2_/NC@TiO_2_||LFP (130 Wh kg^−1^),^[^ ^38^ ^]^ and TiO_2_‐B||LFP (120.8 Wh kg^−1^).^[^ ^39^ ^]^ The cycling performance of both full batteries under the XFC condition (the current density of 10 C corresponds to ≈6 min charging time) was also investigated (Figure 7g). After 2000 cycles, the discharge capacity of the TNO_−_ *_x_* @N‐G||LFP cell maintains at 169.9 mAh g^−1^ with the excellent capacity retention of 86.3%, whereas the TNO||LFP cell retains only 90.8 mAh g^−1^ of capacity and 57.6% of capacity retention. Furthermore, the average discharge voltage after 2000 cycles (≈1.65 V) is close to the original one after five cycles (≈1.67 V), demonstrating the excellent stability of electrochemical interface and redox reactions (Figure S19, Supporting Information).

**Figure 8:**
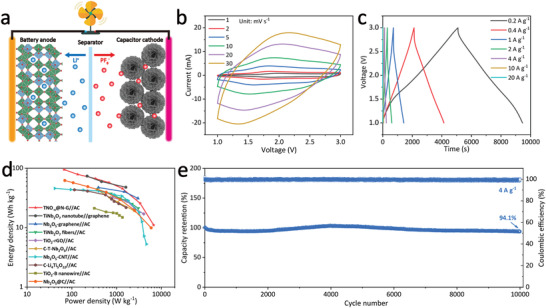
a) Schematic illustration of the charging and discharging process of the TNO_−_ *_x_* @N‐G//AC HLIC. b) CV curves of the TNO_−_ *_x_* @N‐G//AC HLIC at different scan rates. c) Galvanostatic charge–discharge profiles of the TNO_−_ *_x_* @N‐G//AC HLIC at different current densities. d) Ragone plots of the TNO_−_ *_x_* @N‐G//AC HLIC compared with other HLICs by using Ti/Nb‐based materials as the anode. e) Long‐term cycling stability of the TNO_−_ *_x_* @N‐G//AC HLIC at 4 A g^−1^.

Due to the inherent low electronic conductivity of LFP, the TNO_−_ *_x_* @N‐G||LFP lithium‐ion full battery may limit the high‐rate performance of TNO_−_ *_x_* @N‐G anode. Thus, we also designed the TNO_−_ *_x_* @N‐G//AC hybrid lithium‐ion capacitor (HLIC) by using the active carbon electrode as cathode (**Figure** **8**a). During the charging process, PF_6_^−^ anions will be adsorbed on the surface of AC, while Li^+^ cations will be inserted into the crystalline host of TNO_−_ *_x_* @N‐G; the reverse occurs during the discharging process. The fast non‐faradic charge storage mechanism in the AC cathode side is expected to exert the excellent rate capability of TNO_−_ *_x_* @N‐G anode ultimately. CV curves of the TNO_−_ *_x_* @N‐G//AC HLIC at different scan rates ranging from 1 to 30 mV s^−1^ are shown in Figure 8b, which are faintly varied from a rectangular shape for an ideal supercapacitor shape due to the combination of intercalation‐type charge storage processes at the anode. With the stepwise increase of scan rate, the reduction peak and oxidation peak gradually shift to more negative and more positive positions, respectively, implying the small electrochemical polarization. Moreover, the galvanostatic charge–discharge profiles of the TNO_−_ *_x_* @N‐G//AC HLIC are also slightly derived from a linear shape (Figure 8c). The good symmetry of voltage profiles and high CE during the charge/discharge processes demonstrates its high reversibility and low capacity losses. It should be noticed that the TNO_−_ *_x_* @N‐G//AC HLIC can keep working at the extremely high current density of 20 A g^−1^ (corresponding to achieve a complete cycle within 12 s), confirming the good fast charge/discharge characteristic. Ragone plots of the TNO_−_ *_x_* @N‐G//AC HLIC compared with other HLICs by using Ti/Nb‐based materials as the anode are shown in Figure 8d. The TNO_−_ *_x_* @N‐G//AC HLIC can deliver a high energy density of 95.1 Wh kg^−1^. And a high power density of 6722.7 W kg^−1^ is achieved with the energy density retention of 11.1 Wh kg^−1^, which are superior than other HLICs including TiNb_2_O_7_ nanotube//graphene,^[^ ^40^ ^]^ Nb_2_O_5_‐graphene//AC,^[^ ^41^ ^]^ TiNb_2_O_7_ fibers//AC,^[^ ^42^ ^]^ TiO_2_‐rGO//AC,^[^ ^43^ ^]^ C‐T‐Nb_2_O_5_//AC,^[^ ^44^ ^]^ Nb_2_O_5_‐CNT//AC,^[^ ^45^ ^]^ C‐Li_4_Ti_5_O_12_//AC,^[^ ^46^ ^]^ TiO_2_‐B nanowire//AC,^[^ ^47^ ^]^ and Nb_2_O_5_@C//AC.^[^ ^48^ ^]^ Most importantly, the capacity retention of the TNO_−_ *_x_* @N‐G//AC HLIC reaches as high as 94.1% with a high average CE of nearly 100% after 10 000 cycles at 4 A g^−1^ (Figure 8e).

## 3 Conclusion

In conclusion, we rationally designed and synthesized the TNO_−_ *_x_* @N‐G composed of nanoporous TNO encapsulated in conductive N‐doped graphene matrix assisted with the simple solution‐combustion and electrostatic self‐assembly approach. Meanwhile, the reducing environment provided by the H_2_ and neighboring graphene also leads to the formation of oxygen defects in the crystalline host of TNO. DFT calculation results indicate that the successful introduction of oxygen defects in TNO can not only reduce the Li^+^ diffusion energy barrier but also enhance its intrinsic electronic conductivity. EIS and power electronic conductivity measurements validate that the synergistic enhancement of electron/ion transport endows the TNO_−_ *_x_* @N‐G facile electrochemical kinetics. Consequently, the TNO_−_ *_x_* @N‐G can achieve a considerable capacity of 89.2 mAh g^−1^ even at the ultimately high current density of 100 C. Moreover, after 2000 cycles at 10 C, the TNO_−_ *_x_* @N‐G exhibited a higher reversible capacity of 199.0 mAh g^−1^ and a higher capacity retention of 86.5% than those of pristine TNO (155.8 mAh g^−1^, 59.4%). By in situ XRD and Raman spectroscopy, the structural reversibility and a possible “structural activation” process in the first discharging process of the TNO_−_ *_x_* @N‐G was verified. Most importantly, various electrochemical devices including lithium‐ion full battery and hybrid lithium‐ion capacitor by using the TNO_−_ *_x_* @N‐G electrode as anode showed remarkable rate capability and excellent cycling stability, verifying its compatibility in practical applications.

## Conflict of Interest

The authors declare no conflict of interest.

## Supporting information

Supporting Information

## Data Availability

Research data are not shared.
