# Supplementary material for: Synchronous Manipulation of Ion and Electron Transfer in Wadsley–Roth Phase Ti‐Nb Oxides for Fast‐Charging Lithium‐Ion Batteries
Source: Adv Sci (Weinh). 2021 Dec 28;9(6):2104530. doi: 10.1002/advs.202104530 (PMC8867197; doi:10.1002/advs.202104530)
Supplement: Supplementary file 1 — Supporting Information [file ADVS-9-2104530-s001.pdf]

ADVANCED SCIENCE
Open Access

Supporting Information

for *Adv. Sci.*, DOI: 10.1002/advs.202104530

Synchronous manipulation of ion and electron transfer in Wadsley-Roth phase Ti-Nb oxides for fast-charging lithium-ion batteries

Yang Yang,[1] Jingxin Huang,[1] Zhenming Cao,[1] Zeheng Lv,[2] Dongzhen Wu,[1] Zhipeng Wen,[1] Weiwei Meng,[3] Jing Zeng,[1] Cheng Chao Li[2,], Jinbao Zhao[1,*]*

Electronic Supporting Information (ESI)

Synchronous manipulation of ion and electron transfer in Wadsley-Roth phase Ti-Nb oxides for fast-charging lithium-ion batteries

Yang Yang,[1] Jingxin Huang,[1] Zhenming Cao,[1] Zeheng Lv,[2] Dongzhen Wu,[1] Zhipeng Wen,[1] Weiwei Meng,[3] Jing Zeng,[1] Cheng Chao Li[2,*], Jinbao Zhao[1,*]

[1]State Key Lab of Physical Chemistry of Solid Surfaces, State-Province Joint Engineering Laboratory of Power Source Technology for New Energy Vehicle, College of Chemistry and Chemical Engineering, Xiamen University, Xiamen, 361005, P. R. China

[2]School of Chemical Engineering and Light Industry, Guangdong University of Technology, Guangzhou 510006, P. R. China

[3]State Key Laboratory of Vanadium and Titanium Resources Comprehensive Utilization, Panzhihua 617000, P. R. China

AUTHOR INFORMATION

Corresponding Author

*E-mail: licc@gdut.edu.cn; jbzhao@xmu.edu.cn

Experimental

Preparation of TNO nanoparticles

Titanium n-butoxide (Aladdin), ammonia (Xilong Scientific Co., Ltd), nitric acid (Xilong Scientific Co., Ltd), nibobium oxalate hydrate ($Nb(HC_2O_4)_5{\cdot}xH_2O$, Alfa aesar), glycine (Sinopharm Chemical Reagent Co., Ltd), poly (diallyldimethylammonium chloride) (PDDA) aqueous solution (Aladdin, Mw = 100,000-200,000, 20 wt%) and GO aqueous suspension (2 wt%, Shanghai Ashine Technology Development Co., Ltd) were used as received.

TNO nanoparticles were prepared by solution-combustion synthesis using titan nitrate [$TiO(NO_3)_2$] and $Nb(HC_2O_4)_5{\cdot}xH_2O$ as the oxidant precursors and glycine as the fuel. In a typical experiment, 1 mmol $Nb(HC_2O_4)_5{\cdot}xH_2O$ and 20 mmol glycine were added into 0.5 mmol [$TiO(NO_3)_2$] aqueous solution and stirred for 1 h to form a clear solution. Subsequently, the solution was transferred into a 50 mL alumina crucible and put into a preheated muffle furnace at 900 °C maintaining for 1 h to obtain the TNO nanoparticles. The preparation of the [$TiO(NO_3)_2$] aqueous solution was based on a previously reported procedure with minor modifications.[1] Briefly, 4 mL diluted ammonia solution was mixed with 0.17 g titanium n-butoxide (0.5 mmol) under vigorously stirring in ice-water bath to obtain the $TiO(OH)_2$ suspension. Then 1.5 mL nitric acid was added into this suspension to obtain the transparent [$TiO(NO_3)_2$] aqueous solution. Then, 0.65 g $Nb(HC_2O_4)_5{\cdot}xH_2O$ (1 mmol) was added into the solution based on the ratio of Ti:Nb = 1:2 in $TiNb_2O_7$.

Preparation of the TNO_{-x}@N-G

Firstly, 1.5 g GO suspension was dispersed in 30 mL deionized water by sonication for 1 h to form a GO aqueous dispersion. Then 0.3 g TNO nanoparticles and 3 mL PDDA aqueous solution were dispersed in 150 mL deionized water by sonication for 1 h. The white precipitate was collected by centrifugation, washed by 150 mL deionized water and added into 50 mL deionized water to form a TNO-PDDA aqueous dispersion. The TNO-PDDA aqueous dispersion was drop-wise added into the GO aqueous dispersion under stirring. The resulting flocculent suspension was frozen by liquid nitrogen and then freeze-dried, followed by thermal treatment at 700 °C under the H_2/Ar atmosphere with the heat rate of 5 °C min^{-1} to obtain the final TNO_{-x}@N-G.

Characterization

Zeta potentials were measured with the zeta potential analyzer (90 Plus PALS, Brookhaven Instruments Corporation). The crystal structure of the samples was characterized by X-ray diffraction (XRD) on the Rigaku miniflex 600 diffractometer with Cu Kα radiation (λ = 0.154 nm). Raman spectra were collected with the Renishaw inVia Raman microscope using 532 nm Ar-ion laser. The morphology of the samples was characterized by using the high-resolution transmission electron microscopy (HR-TEM, FEI, TECNAI G2 F30) and (JEOL-2100). Thermo gravimetric analysis (TGA) was performed on TGA/DSC 1 $STAR^e$ System (Mettler-Toledo, Switzerland) under air flow (30 ~ 800 ºC, 10 ºC min^{-1}). Powder electronic conductivity tests were performed on the Power Resistivity Meter SZT-D (Suzhou Jingge Electronics Co., Ltd.). Differential scanning calorimetric (DSC) tests

were conducted on the electrochemically lithiated TNO_{-x}@N-G and graphite electrodes to study the thermal stability, which were carried out on Netzsch STA 449 F3 from 30 to 200 °C at a scanning rate of 5 °C min^{-1}.

Electrochemical measurement

The working electrode slurry was prepared by mixing active materials (70 wt%), acetylene black (20 wt%) and bi-component binders of same weight of carboxyl methyl cellulose (CMC) and styrene-butadiene resin (SBR) (10 wt%) using deionized water as the solvent. The slurry was spread on Cu foil with an applicator, and dried for 10 h at 80 °C in a vacuum oven. The loading mass of active materials was 1.3 ~ 1.5 mg cm^{-2}. CR2016 type coin cells were used to measure the electrochemical properties of the samples assembled in an Ar-filled glove box. Li metal was used as the counter electrode and celgard 2400 was used as the separator. The electrolyte was l M $LiPF_6$ dissolved in the mixture of ethylene carbonate (EC) and diethyl carbonate (DEC) with the volumetric ratio of 1: 1. The cells were tested using a charge/discharge unit (Neware BTS battery charger, Shenzhen, China) in the potential range from 1.0 to 3.0 V at various current rates. The theoretical capacity of $TiNb_2O_7$ is calculated to be 232.2 mAh g^{-1} based on one electron transfer per transition metal atom, and 1 C is simplified to be 200 mA g^{-1}. Cyclic voltammetry (CV) tests were conducted on the CHI 1030C electrochemical workstation at the scan rate of 0.1 mV s^{-1}. Electrochemical impedance spectra (EIS) were measured on the Autolab 302N in the frequency range from 100 mHz to 100 kHz.

The $LiFePO_4$ cathode was prepared by mixing active materials (80 wt%), acetylene black (10 wt%) and poly(1,1-difluoroethylene) (PVDF, 10 wt%) using N-methylpyrrolidone (NMP) as the solvent. The slurry was spread on Al foil with an applicator, and dried overnight at 80 °C in a vacuum oven. The active carbon cathode was also fabricated by similar method except that carbon cloth was used as the current collector. For the assembly of CR-2032 type lithium-ion full cells, the TNO and TNO_{-x}@N-G electrodes were used as anode, and the $LiFePO_4$ electrode was used as cathode, respectively. In lithium-ion full cells, the capacity ratio of the negative electrode to positive electrode (N/P ratio) was controlled to be 0.80 ~ 0.90. Hybrid lithium-ion capacitors were also assembled into CR-2032 type coin cells with prlithiated TNO_{-x}@N-G as the anode (pre-cycled in half cells for 10 cycles, and disassembled inside the glove box at a lithiation state at 1.0 V (vs. Li^+/Li), and active carbon electrode as the cathode. In hybrid lithium-ion capacitors, the N/P ratio was carefully controlled to be about 1.

***In-situ* electrochemical XRD and Raman spectroscopy measurement**

The TNO_{-x}@N-G material was coated on the stainless steel grid with the same slurry preparation procedure mentioned above for *in-situ* electrochemical XRD and Raman spectral measurements. The configuration of the *in-situ* electrochemical XRD cell and Raman spectral cell were described in the previous reports.[2] The XRD pattern measurement and the galvanostatic charging and discharging were simultaneously performed in the in-situ electrochemical XRD cell. Every XRD pattern was measured simultaneously with galvanostatic charging and discharging on

Rigaku Ultima IV with the X-ray radiation wavelength of 0.154 nm (Cu Kα), the X-ray radiation power of 1200 W, and the 2θ angle scanning rate at 2° min^{-1} with the step size of 0.02°. The *in-situ* electrochemical XRD test were conducted by a charge/discharge unit (Neware BTS battery charger, Shenzhen, China) in the potential range from 3.0 V to 1.0 at a current density of 20 mA g^{-1}. Every Raman spectral was measured with an acquisition time of 600 s and an interval time of 300 s on Renishaw inVia Raman microscope with the laser wavelength of 532 nm, the laser power of 0.1 mW and the grating of 1800 T.

Ex-situ XRD measurement

The CR2016 type coin cells were assembled as the procedures above. The coin cells were stopped at the lithiation state y=1.6 and 2.0 in the discharging and the charging and the electrodes were taken from the coin cell for the ex-situ XRD measurement with the sealing window. The ex-situ XRD measurement condition is described as follows: the X-ray radiation wavelength is 0.154 nm (Cu Kα), the X-ray radiation power is 1200 W and the 2θ angle scanning rate at 2° min^{-1} with the step size of 0.02° on Rigaku Ultima IV.

Theoretical calculations

All first-principles calculations were performed within the Vienna Ab Initio Simulation Package (VASP) based on density functional theory (DFT).[3] The projector augmented wave (PAW) potentials were used to deal with the electronic exchange-correlation interaction along with the GGA functional in the parameterization of the Perdew Burke and Ernzerhof (PBE) pseudopotential.[4] A

plane wave representation for the wave function with a cut off energy of 450 eV was applied. Geometry optimizations were performed using a conjugate gradient minimization until all the forces acting on the ions were less than 0.02 eV/Å per atom. A 15 Å vacuum in the z direction was used to separate the slabs. In the calculations, a k-point mesh with a spacing of ca. 0.03 $A^{\circ -1}$ was adopted. The long-range interactions (DFT-DF3) are considered as a correction in all the calculation. In addition, for the Ti and Nb atoms, the U schemes need to be applied, and the U has been set as 5.0 and 4.0 eV.

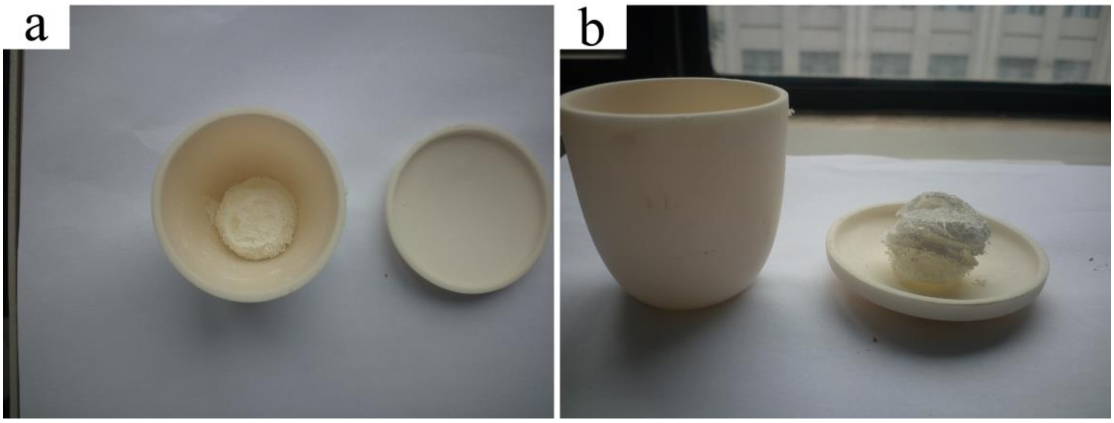

Figure 1. Photographs of the as-prepared TNO through solution combustion method.

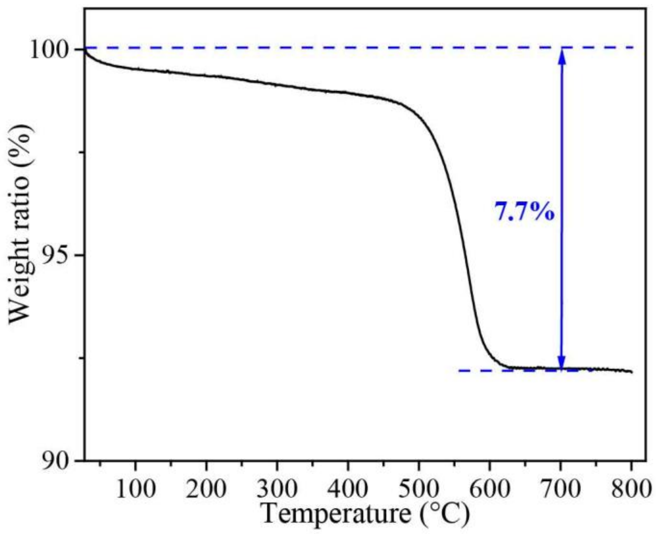

Figure S2. TGA curve of the TNO_{-x}@N-G composite.

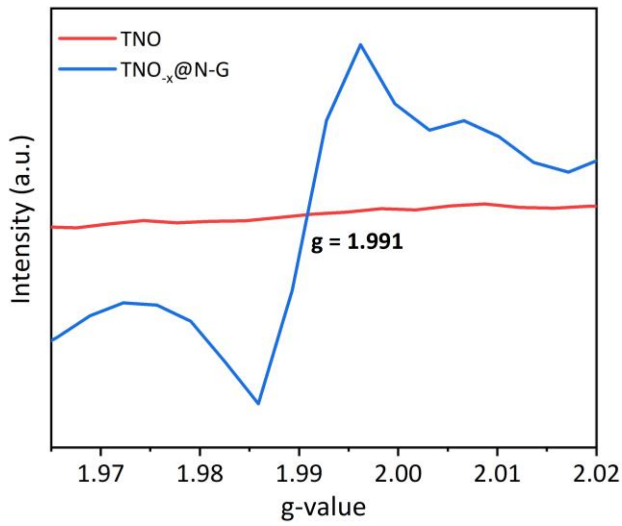

Figure S3. EPR spectra of the TNO and TNO_{-x}@N-G.

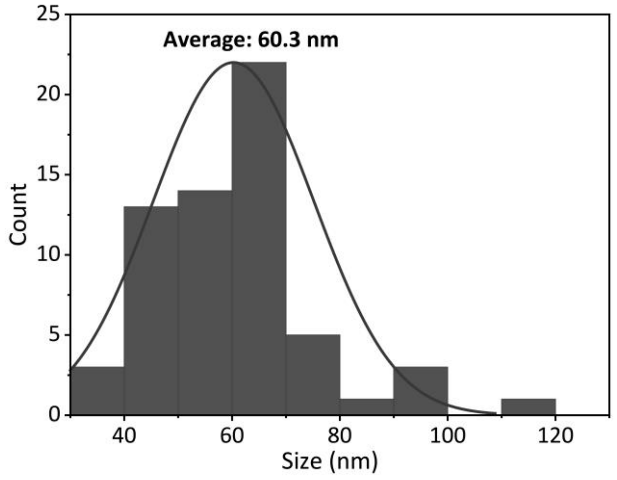

Figure S4. Size distribution of the as-prepared TNO obtained by analyzing the TEM image through the ImageJ software.

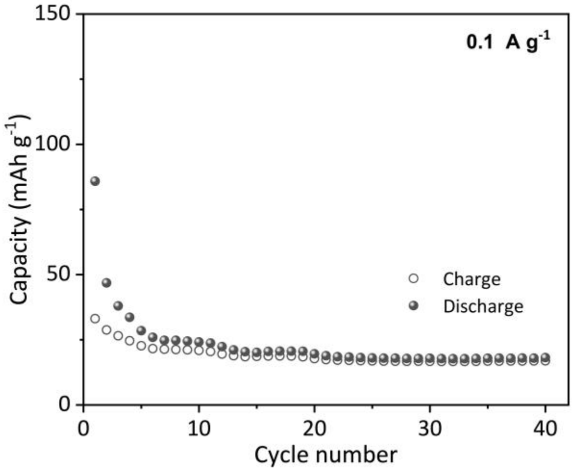

Figure S5. Cycling performance of the graphene electrode at 0.1 A g^{-1} between 1.0 and 3.0 V (vs. Li^+/Li).

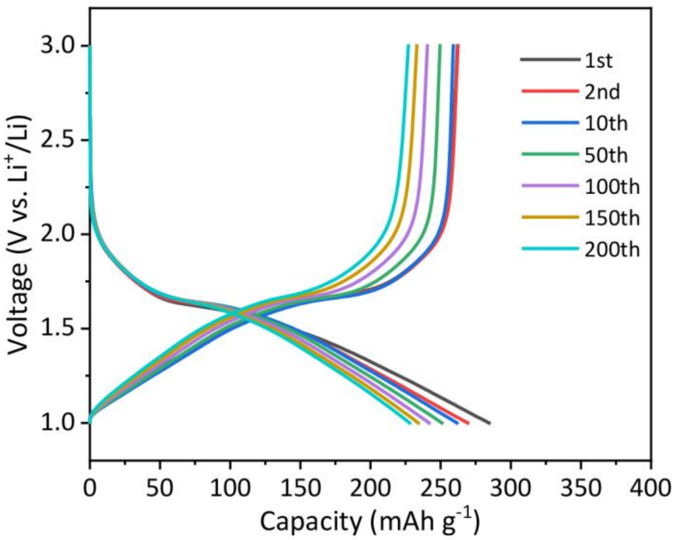

Figure S6. Typical charge and discharge profiles of the TNO at 0.5 C

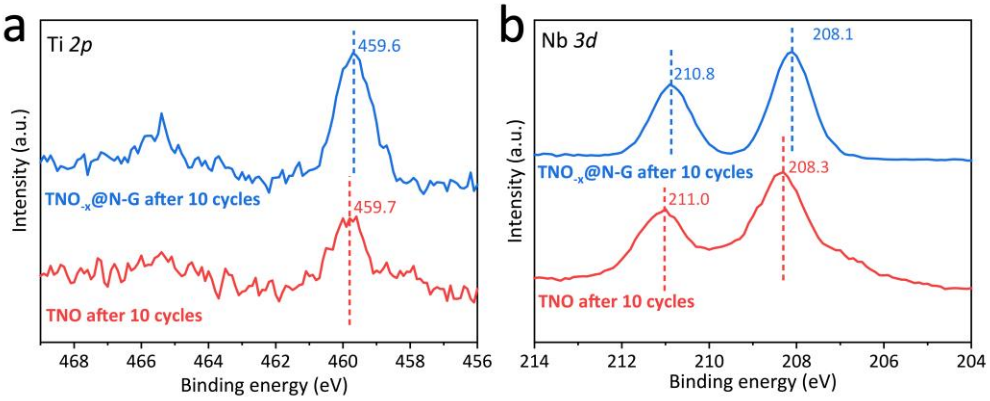

Figure S7. High resolution XPS spectra of the TNO and TNO-x@N-G electrodes after 10 cycles: (a) Ti *2p* and (b) Nb *3d*.

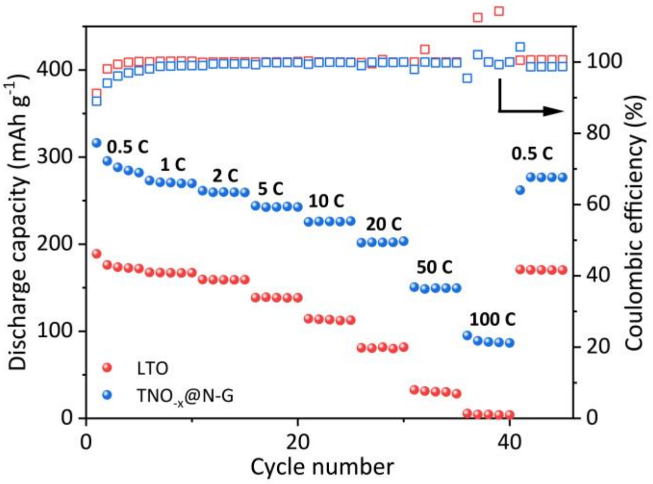

Figure S8. Rate performance of commercial LTO and the TNO_{-x}@N-G.

Table S1. The comparison of the TNO-x@N-G and other previously reported $TiNb_2O_7$-based materials in terms of cycling stability and lifespan.

References	Capacity retention
Carbon Coated Porous Titanium Niobium Oxides as Anode Materials of Lithium-Ion Batteries for Extreme Fast Charge Applications[5]	80.6% after 500 cycles at 6 C
Coarse-grained reduced Mo Ti1−Nb2O7+ anodes for high-rate lithium-ion batteries[6]	73% after 500 cycles at 10 C
Interstitial and substitutional V5+-doped TiNb2O7 microspheres: A novel doping way to achieve high-performance electrode[7]	71.7% after 2000 cycles at 10 C
Ionic Liquid-Directed Nanoporous TiNb2O7 Anodes with Superior Performance for Fast-Rechargeable Lithium-Ion Batteries[8]	74% after 1000 cycles at 5 C
Multiscale Designed Niobium Titanium Oxide Anode for Fast Charging Lithium Ion Batteries[9]	78.3% after 100 cycles at 0.3 C
Nanoscale assembling of graphene oxide with electrophoretic deposition leads to superior percolation network in Li-ion electrodes: TiNb2O7/rGO composite anodes[10]	81.1% after 150 cycles at 0.5 C
Superior performance of ordered macroporous TiNb2O7 anodes for lithium ion batteries: Understanding from the structural and pseudocapacitive insights on achieving high rate capability[11]	82% after 1000 cycles at 10 C
TiNb2O7 hollow nanofiber anode with superior electrochemical performance in rechargeable lithium ion batteries[12]	80.6% after 900 cycles at 10 C
TiNb2O7 nano-particle decorated carbon cloth as flexible self-support anode material in lithium-ion batteries[13]	81% after 500 cycles at 0.5 C
Controlled fabrication and performances of single-core/dual-shell hierarchical structure m-TNO@TiC@NC anode composite for lithium-ion batteries[14]	64.8% after 200 cycles at 1 C
Our work	86.5% after 2000 cycles at 10 C

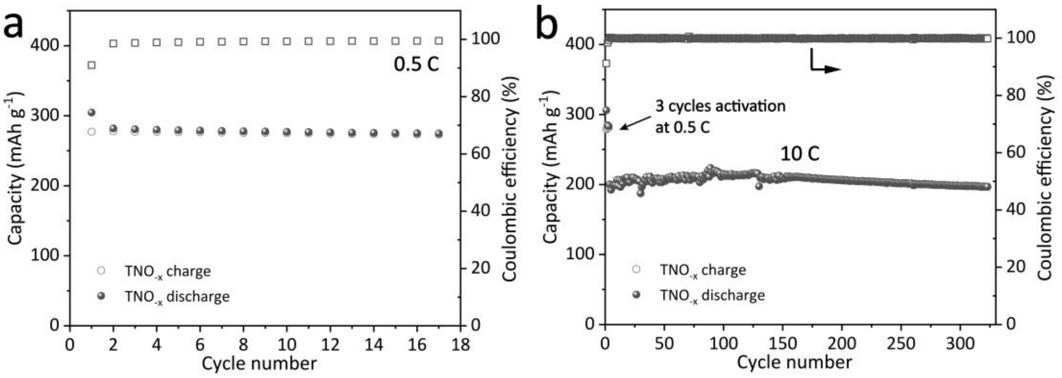

Figure S9. Specific capacity of the TNO_{-x} electrode (a) at 0.5 C and (b) at 10 C.

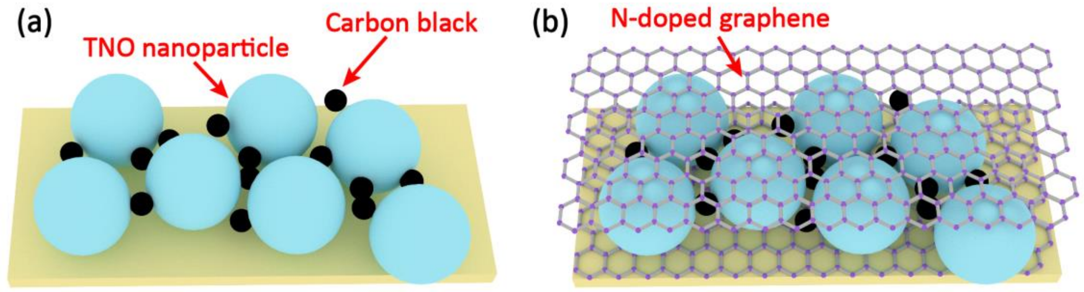

Figure S10. Schematics of electrode configurations of the TNO and TNO_{-x}@N-G electrodes.

It is generally accepted that ensuring the unobstructed electrical connection between active materials to form an integral conductive network in the electrode is crucial for achieving good cycling stability. For the TNO electrode, nanosized carbon black particles are filled in the interstices between TNO nanoparticles, which form a conductive network based on the point contact (Figure S9a). In addition to the point contact conductive mechanism, N-doped graphene layers also serve as 2-D conductive matrix based on the face-to-face contact for the TNO_{-x}@N-G electrode (Figure S9b). The well-established multidimensional conductive framework in the TNO_{-x}@N-G electrode will reduce the charge-transfer resistance and improve the cycling stability especially at high-rate conditions.

Table S2. the kinetic parameters from EIS measurements and powder electronic conductivity for the TNO and TNO_{-x}@N-G.

Sample	R_{sf} (Ω)	$R_{ct\ Li}$ (Ω)	Electronic conductivity (S cm^{-1})
TNO-rGO	19.39	50.61	1.38
TNO	40.41	54.63	3.73×10^{-5}

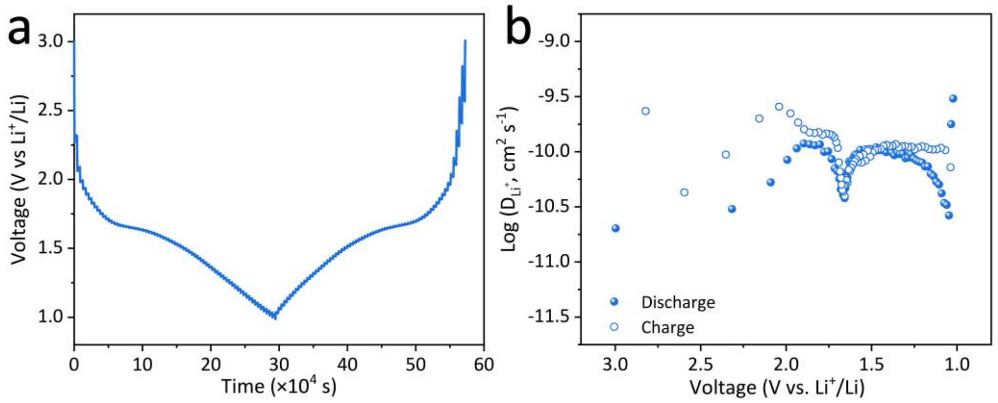

Figure S11. (a) GITT curves and (b) the calculated Li^+ diffusion coefficient (D_{Li^+}) of the TNO_{-x}@N-G electrode.

Table S3. The comparison of D_{Li^+} between the TNO_{-x}@N-G and previously reported $Li_4Ti_5O_{12}$ and TiO_2.

Samples	D_{Li^+} ($cm^2\ s^{-1}$)
Carbon-Coated TiO_2 Nanocrystal[15]	10^{-10}-$10^{-11.2}$
Spindle TiO_2 nanograin[16]	10^{-11}
rGO supported TiO_2 (B)[17]	10^{-14}
$Li_4Ti_5O_{12}$[18]	10^{-16}
N-doped $Li_4Ti_5O_{12}$/C hybrid[18]	10^{-13}
$Li_4Ti_5O_{12}$[19]	10^{-13}
GO/$Li_4Ti_5O_{12}$[19]	10^{-12}-10^{-13}
$Li_4Ti_5O_{12}$[20]	10^{-12}
PANI@$Li_4Ti_5O_{12}$[20]	10^{-12}
$Li_4Ti_5O_{12}$@CN composite[21]	10^{-11}
This work	$10^{-9.5}$-$10^{-10.5}$

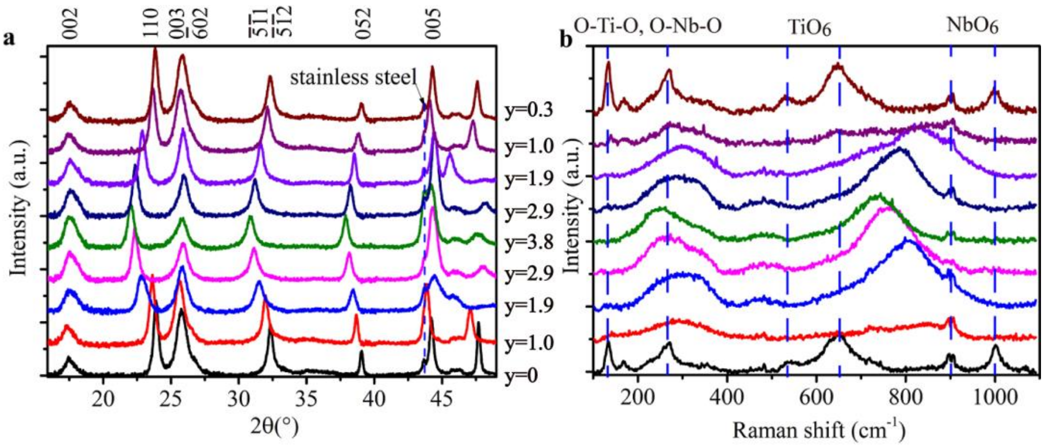

Figure S12. Some single XRD patterns and Raman spectra selected from the Figure 8(b) and (c) for the $Li_yTiNb_2O_7$ (y=0, 1.0, 1.9, 2.9, 3.8 in the discharging and y= 2.9, 1.9, 1.0, 0.3 in the charging).

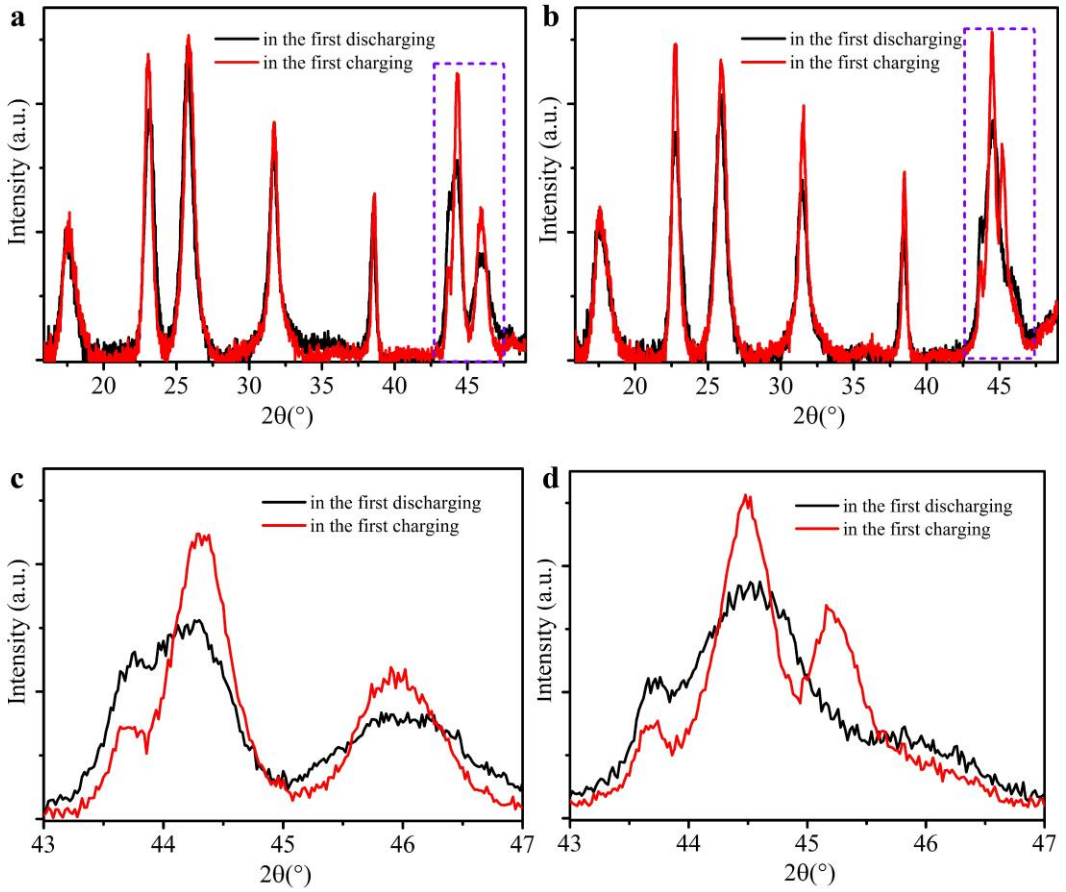

Figure S13. Comparison of the XRD pattern in the first discharging and charging for $Li_yTiNb_2O_7$ with (a) y=1.6 and (b)y=2.0. The purple dashed rectangles indicate the same 2 angle range with that indicated by the red dashed rectangles in the Figure 8(b). (c) and (d) correspond to the purple dashed rectangles in (a) and (b).

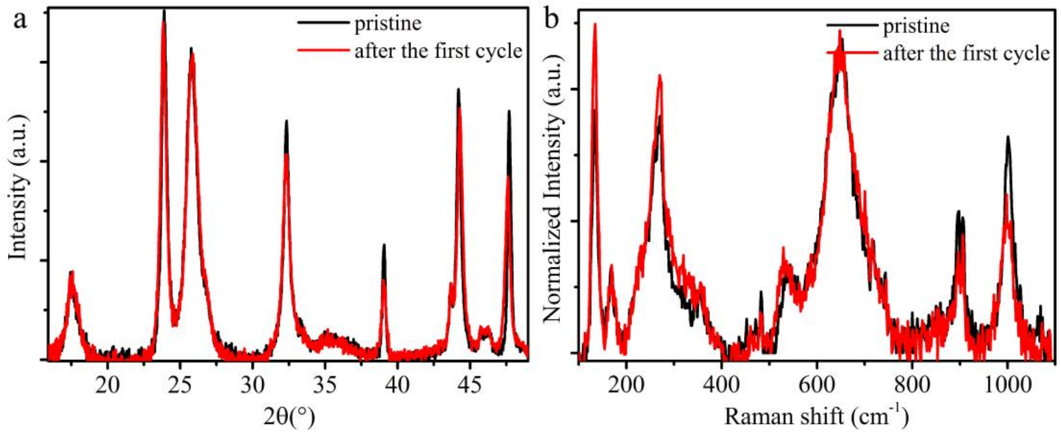

Figure S14. (a) The pristine XRD pattern and the XRD pattern after the discharging/charging cycle, (b) the pristine Raman spectra and the Raman spectra after the discharging/charging cycle which are normalized as the intensity of the peaks at 650 cm^{-1}.

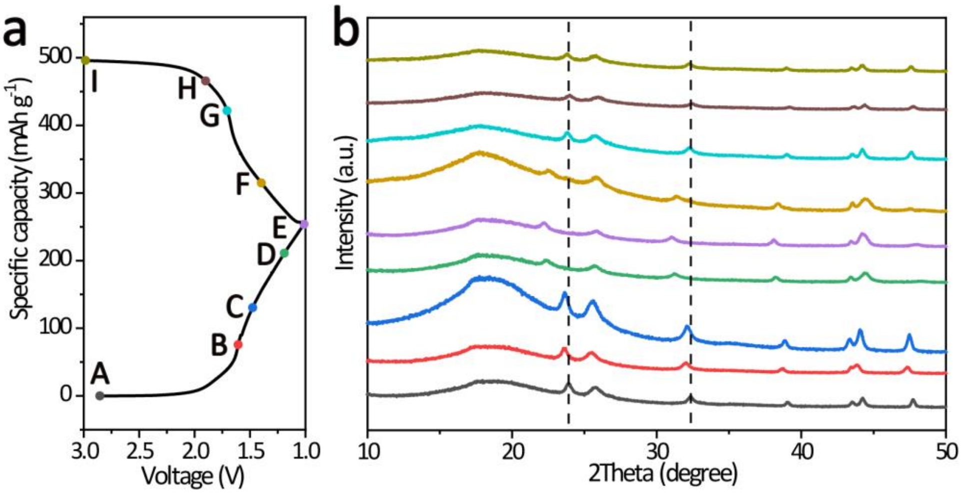

Figure S15. (a) Galvanostatic charge-discharge curves and (b) *ex-situ* XRD patterns of the TNO electrode.

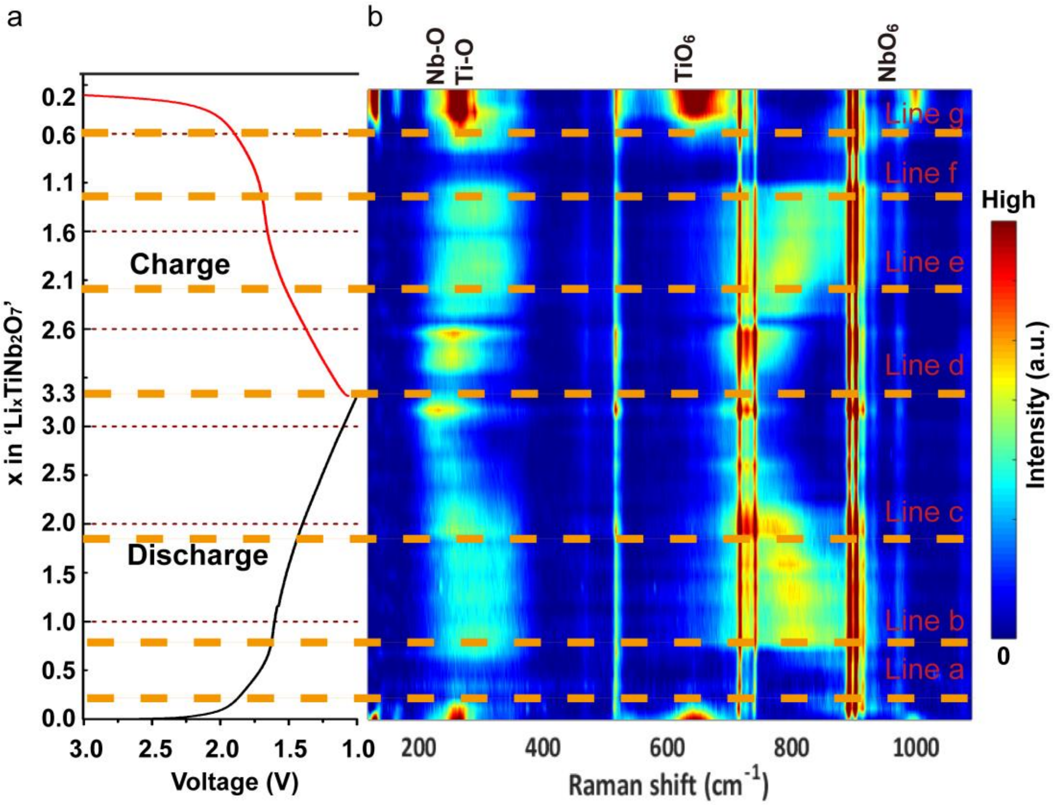

Figure S16. *In-situ* electrochemical Raman spectra of the TNO electrode: (a) the voltage curves in the first electrochemical cycling of the TNO in the *in-situ* electrochemical Raman spectroscopic cell at 30 mA g^{-1} (the x in '$Li_xTiNb_2O_7$' is calculated based on the $TiNb_2O_7$ quantity in the TNO); (b) the simultaneous *in-situ* electrochemical Raman spectra corresponding to (a) with the intensity colorbar from 0 to 2000 cps on the right. The horizontal dashed lines in (a) and (b) indicate the different reaction stages of TNO in the initial discharging/charging processes.

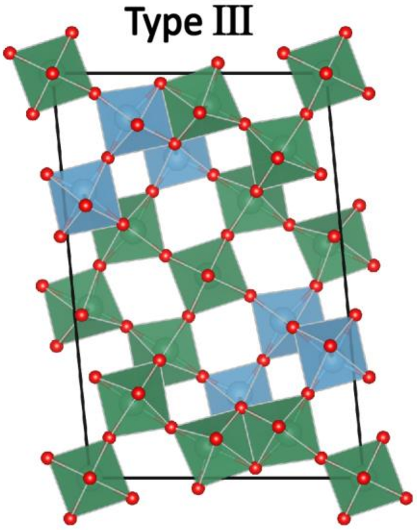

Figure S17. (a) The type III crystal structure model of $TiNb_2O_7$.

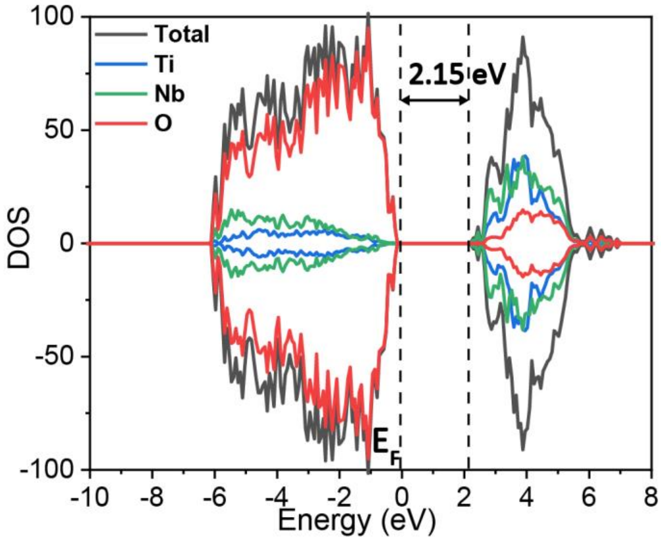

Figure S18. DOS of pure $TiNb_2O_7$.

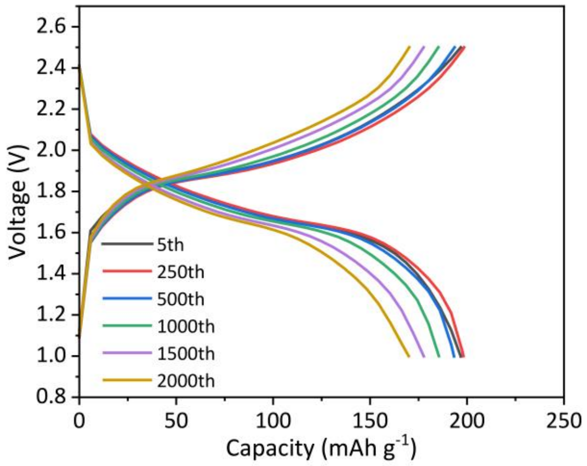

Figure S19. Galvanostatic charge-discharge profiles of the TNO_{-x}@N-G||LFP cell at 10 C.

References

[1] a) X. Li, H. C. Lin, W. J. Cui, Q. Xiao, J. B. Zhao, *ACS Appl. Mater. Interfaces* **2014**, 6, 7895; b) A. S. Prakash, P. Manikandan, K. Ramesha, M. Sathiya, J. M. Tarascon, A. K. Shukla, *Chem. Mater*.**2010**, 22, 2857.

[2] Y. Yang, J. Huang, J. Zeng, J. Xiong, J. Zhao, *ACS Appl. Mater. Interfaces* **2017**, 9, 32801.

[3] J. P. Perdew, K. Burke, M. Ernzerhof, *Phys. Rev. Lett.* **1996**, 77, 3865.

[4] a) Kresse, Furthmuller, *Phys. Rev. B, Condensed matter* **1996**, 54, 11169; b) G. Kresse, D. Joubert, *Phys. Rev. B* **1999**, 59, 1758.

[5] H. Lyu, J. Li, T. Wang, B. P. Thapaliya, S. Men, C. J. Jafta, R. Tao, X.-G. Sun, S. Dai, *ACS Appl. Energy Mater.* **2020**, 3, 5657.

[6] L. Zhao, S. Wang, Y. Dong, W. Quan, F. Han, Y. Huang, Y. Li, X. Liu, M. Li, Z. Zhang, J. Zhang, Z. Tang, J. Li, *Energy Storage Mater.* **2021**, 34, 574.

[7] K. Liu, J.-a. Wang, J. Yang, D. Zhao, P. Chen, J. Man, X. Yu, Z. Wen, J. Sun, *Chem. Eng. J.* **2021**, 407, 127190.

[8] R. Tao, G. Yang, E. C. Self, J. Liang, J. R. Dunlap, S. Men, C. L. Do- Thanh, J. Liu, Y. Zhang, S. Zhao, H. Lyu, A. P. Sokolov, J. Nanda, X. G. Sun, S. Dai, *Small* **2020**, 16, 2001884.

[9] T. Tian, L. L. Lu, Y. C. Yin, F. Li, T. W. Zhang, Y. H. Song, Y. H. Tan, H. B. Yao, *Adv. Funct. Mater.* **2021**, 31, 2007419.

[10] M. Uceda, H.-C. Chiu, J. Zhou, R. Gauvin, K. Zaghib, G. P. Demopoulos, *Nanoscale* **2020**, 12, 23092.

[11] S. Lou, X. Cheng, Y. Zhao, A. Lushington, J. Gao, Q. Li, P. Zuo, B. Wang, Y. Gao, Y. Ma, C. Du, G. Yin, X. Sun, *Nano Energy* **2017**, 34, 15.

[12] H. Yu, H. Lan, L. Yan, S. Qian, X. Cheng, H. Zhu, N. Long, M. Shui, J. Shu, *Nano Energy* **2017**, 38, 109.

[13] J. Luo, J. Peng, P. Zeng, Z. Wu, J. Li, W. Li, Y. Huang, B. Chang, X. Wang, *Electrochim. Acta* **2020**, 332, 135469.

[14] S. Yoon, S.-Y. Lee, N. Tuan Loi, I. T. Kim, S.-G. Woo, K. Y. Cho, *J. Alloys Compd.* **2018**, 731, 437.

[15] J. U. Ha, J. Lee, M. A. Abbas, M. D. Lee, J. Lee, J. H. Bang, *ACS Appl. Mater. Interfaces* **2019**, 11, 11391.

[16] C. Guo, Q. Tian, L. Yang, *J. Alloys Compd.* **2019**, 776, 740.

[17] J. He, J. Yang, J. Jiang, M. Xu, Q. Wang, *J. Alloys Compd.* **2021**, 853, 157330.

[18] K. Zhou, X. Fan, W. Chen, F. Chen, X. Wei, A. Li, J. Liu, *Electrochim. Acta* **2017**, 247, 132.

[19] B. Rajagopalan, B. Kim, S. H. Hur, I.-K. Yoo, J. S. Chung, *J. Alloys Compd.* **2017**, 709, 248.

[20] L. Mo, H. Zheng, *Energy Rep.* **2020**, 6, 2913.

[21] H. Xiao, X. Huang, Y. Ren, X. Ding, S. Zhou, *Front. Chem.* **2019**, 7.
